# The inhibitory receptor Siglec‐G controls the severity of chronic lymphocytic leukemia

**DOI:** 10.15252/embr.202256420

**Published:** 2023-07-10

**Authors:** Bettina Röder, Hannah Fahnenstiel, Simon Schäfer, Bettina Budeus, Maria Dampmann, Melanie Eichhorn, Sieglinde Angermüller, Claudia Brost, Thomas H Winkler, Marc Seifert, Lars Nitschke

**Affiliations:** ^1^ Division of Genetics, Department of Biology University of Erlangen Erlangen Germany; ^2^ Medical Faculty, Institute of Cell Biology (Cancer Research) University of Duisburg‐Essen Essen Germany; ^3^ Department of Hematology and Stem Cell Transplantation University Hospital Essen Essen Germany

**Keywords:** BCR signaling, CLL, Siglec‐10, Siglec‐G overexpressing mice, Siglecs, Cancer, Immunology, Signal Transduction

## Abstract

Chronic Lymphocytic Leukemia (CLL) is the most common leukemia in adults in the Western world. B cell receptor (BCR) signaling is known to be crucial for the pathogenesis and maintenance of CLL cells which develop from mature CD5^+^ B cells. BCR signaling is regulated by the inhibitory co‐receptor Siglec‐G and Siglec‐G‐deficient mice have an enlarged CD5^+^ B1a cell population. Here, we determine how Siglec‐G expression influences the severity of CLL. Our results show that Siglec‐G deficiency leads to earlier onset and more severe course of the CLL‐like disease in the murine Eμ‐TCL1 model. In contrast, mice overexpressing Siglec‐G on the B cell surface are almost completely protected from developing CLL‐like disease. Furthermore, we observe a downmodulation of the human ortholog Siglec‐10 from the surface of human CLL cells. These results demonstrate a critical role for Siglec‐G in disease progression in mice, and suggest that a similar mechanism for Siglec‐10 in human CLL may exist.

## Graphical abstract

Deficiency of the inhibitory receptor Siglec‐G in mice leads to earlier onset and more severe chronic lymphocytic leukemia (CLL). In contrast, overexpression of Siglec‐G on B cells protects mice from CLL.

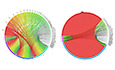

## Introduction

Chronic lymphocytic leukemia (CLL) is the most common leukemic disease in adults in the Western world and typically occurs in elderly patients. CLL is characterized by a monoclonal expansion of mature CD5^+^ B cells in lymphoid tissue, bone marrow, and peripheral blood. Despite multiple treatment options (Hallek & Furstenau, 2019), CLL remains an incurable disease, with a clinical course that is highly variable and difficult to predict. The cellular origin, in particular the germinal center (GC) experience during the early phase of CLL pathogenesis proved to be a powerful clinical predictor (Oakes *et al*, 2016). CLL cases with an immunoglobulin heavy‐chain variable gene (IgV_H_) mutation frequency below 2% show inferior outcome (Hamblin *et al*, 1999). This led to the definition of two major CLL subsets, expressing an either unmutated (uCLL) or mutated (mCLL) immunoglobulin (Ig)‐rearrangement, with putative derivation from pre‐GC and post‐GC B cell subsets, respectively (Bosch & Dalla‐Favera, 2019). Molecular comparison of transcriptome analysis narrowed down the cellular origin of CLL to normal CD5^+^ B cell subsets with either pre‐GC (CD5^+^ naive) or post‐GC (CD5^+^CD27^+^ memory) differentiation stage (Seifert *et al*, 2012).

About 30% of CLL cases are characterized by homologous Ig‐rearrangements, suggesting selection of the B‐cell receptor (BCR) by similar antigens (Chiorazzi & Ferrarini, 2003; Stamatopoulos *et al*, 2007). These patients with CLL cells expressing so‐called “stereotyped” B‐cell receptors suffer from a more aggressive course of the disease and worse outcome regardless of Ig‐mutation status (Rai & Jain, 2016). CLL cells often express self‐aggregating BCR sequences that can elicit ligand‐independent, autonomous BCR signaling (Duhren‐von Minden *et al*, 2012; Minici *et al*, 2017). The importance of BCR signaling for development and maintenance of CLL cells has recently been shown in a mouse model (Schmid *et al*, 2022). Furthermore, this led to the clinical development of inhibitors for cellular kinases, which are downstream of BCR signaling, as therapeutic targets in CLL. Especially Ibrutinib, an inhibitor for Bruton's tyrosine kinase (BTK), has been approved and is widely used as a successful therapy against CLL (Burger & Chiorazzi, 2013; Hallek & Furstenau, 2019). The BCR complex is crucial for the survival of peripheral B cells and its signaling is closely regulated by plasma membrane co‐receptors and intracellular signaling proteins. While several intracellular kinases seem to be crucial for CLL survival, the role of inhibitory proteins is less investigated. When one of two inhibitory phosphatases for the crucial PI3K B‐cell survival pathway, SHIP1 or PTEN, were inhibited or deleted, impaired survival of CLL cells or worse CLL outcome were reported in mouse models, respectively (Ecker *et al*, 2021; Schmid *et al*, 2022). The role of B‐cell inhibitory receptors has hardly been studied for CLL development so far.

Our group works on B‐cell inhibitory receptors of the sialic‐acid binding immunoglobulin‐like lectin (Siglec) family. B cells express the inhibitory receptor Siglec‐G in the mouse, or its human ortholog Siglec‐10 (Meyer *et al*, 2018). We have in the past studied the role of Siglec‐G by genetic mouse models. Siglec‐G‐deficient mice show a 5‐ to 10‐fold expansion of the CD5^+^ B‐cell population, which are B1a cells, B cells with specific functions in the mouse (Baumgarth, 2011). In Siglec‐G‐deficient B1a cells enhanced BCR‐induced Ca^2+^ signaling was observed, which is crucial for the maintenance of this cell type (Hoffmann *et al*, 2007). Furthermore, Siglec‐G‐deficient mice develop autoantibodies and signs of lupus‐like autoimmune disease upon aging (Bökers *et al*, 2014; Müller & Nitschke, 2014; Müller *et al*, 2015). Murine CD5^+^ B1a cells share features of human CD5^+^ CLL cells in some aspects, including expression of a restricted Ig repertoire using similar IgV_H_ families, including specificities for autoantigens and oxidized phospholipids (Herve *et al*, 2005; Baumgarth, 2011). It was therefore of interest to investigate whether the largely increased CD5^+^ B1a cell population and the higher BCR signaling in B1a cells of Siglec‐G‐deficient (*Siglecg* ^−/−^) mice might promote or enhance the development of CLL‐like disease in mice. Previously, in aging *Siglecg* ^−/−^ animals, the development of heterogenous B‐cell lymphoid tumors could be observed, but interestingly no spontaneous CLL developed (Simonetti *et al*, 2014). We decided to analyze the role of Siglec‐G in murine CLL development by crossing Siglec‐G‐deficient mice into the commonly used Eμ‐TCL1 transgenic CLL mouse model (Bichi *et al*, 2002). TCL1 is the T‐cell leukemia‐1 gene, an oncogene which is commonly activated by translocations in T‐cell leukemias and is also activated in several B‐cell leukemias (Teitell, 2005). In this mouse line TCL1 is expressed by a B‐cell specific promoter and enhancer (Bichi *et al*, 2002). Eμ‐TCL1 transgenic mice develop a lymphoproliferative disorder of CD5^+^ B cells starting at about 6 months of age and leading to a CLL‐like disease at the age of 10–12 months with initially oligoclonal and then monoclonal leukemic B‐cell populations. The BCR sequences of CLL‐like cells in Eμ‐TCL1 transgenic mice are barely Ig‐mutated and resemble human uCLL cells with restricted IgV_H_ repertoire and specificities for autoantigens and microbial antigens (Yan *et al*, 2006).

Our results show that Siglec‐G deficiency leads to earlier onset and more severe course of the CLL‐like disease in Eμ‐TCL1 transgenic mice. To investigate whether Siglec‐G overexpression has an opposite effect on CLL development, we generated a mouse line where the Siglec‐G expression level on the B cell surface is about five times higher than in wild type (WT) mice. Interestingly, the overexpression of this inhibitory receptor could almost completely prevent development of the CLL‐like disease in Eμ‐TCL1 transgenic mice. Furthermore, we observed a downmodulation of the human orthologue Siglec‐10 from the surface of human CLL cells. These results show that Siglec‐G in the mouse is a crucial regulator of CLL severity and a parallel mechanism for Siglec‐10 in human CLL may exist.

## Results

### Earlier and stronger expansion of the CLL‐like population in the blood of TCL1 × Siglecg^−/−^ mice

**Figure 1:**
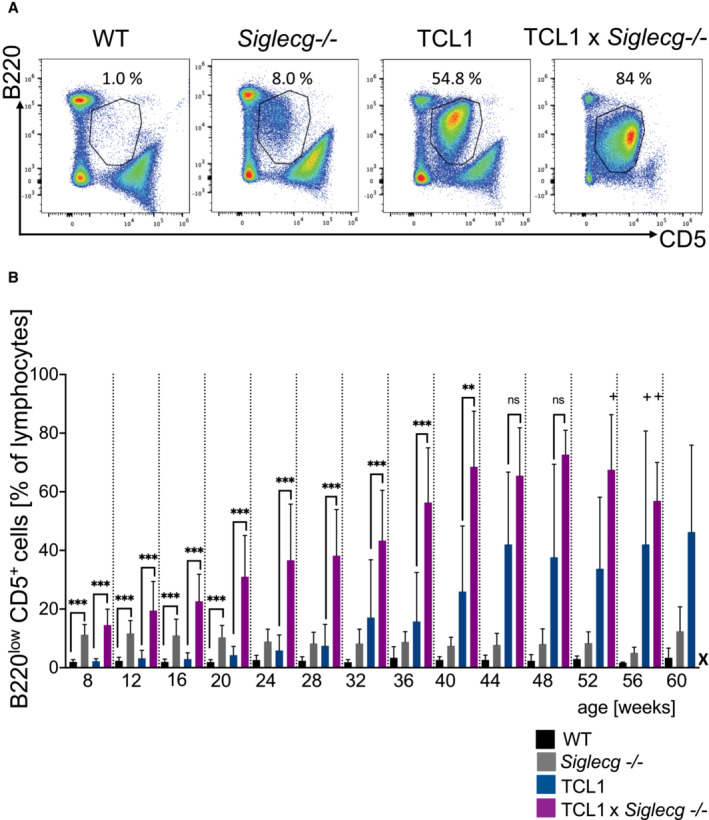
Earlier and stronger expansion of CLL‐like cells in the blood of TCL1 × *Siglecg* ^−/−^ mice To monitor the progression of the CLL‐like population, blood was collected from the animals every 4 weeks up to an age of 60 weeks and analyzed by flow cytometry. Representative FACS plots show examples of stainings of B220^low^ CD5^+^ B cells. (CLL‐like cells) and the selected gate for (B).The diagram displays the percentage of CLL‐like cells in relation to all lymphocytes over a period of 60 weeks. Shown are mean values with ± SD. Significant differences between groups were determined by one‐way ANOVA with Kruskal–Wallis test (no normal distribution) and corrected for multiple comparison with Dunn's test, **P < 0.01, ***P < 0.001. Summarized data from more than 40 independent experiments for individual time points, comprising *n* = 4–75 animals per time point and genotype. X = no surviving TCL1 × *Siglecg* ^−/−^ animals at the age of 60 weeks. + indicates that in these groups only 4 animals were used for the analysis and therefore no calculation of significance was possible. Source data are available online for this figure.

**Figure EV1:**
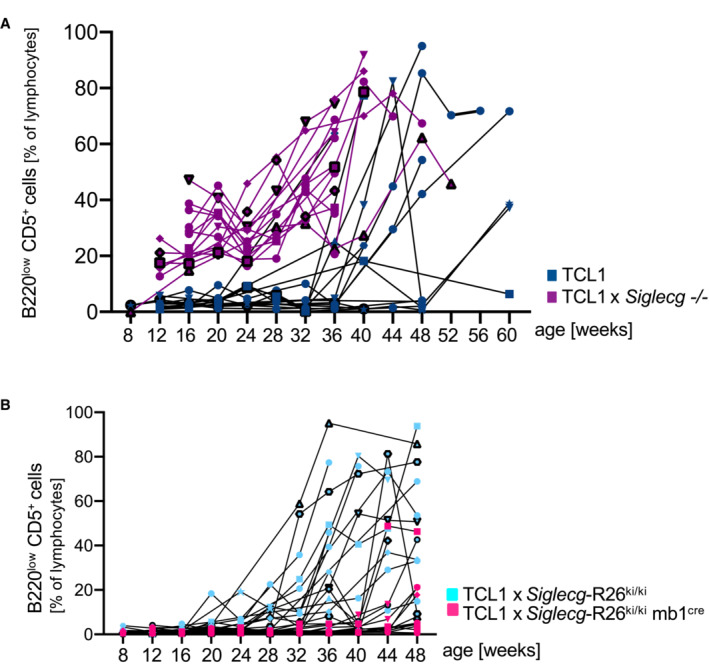
Earlier expansion of the CLL‐like cell population in TCL1 × Siglecg^−/−^ mice and later expansion in TCL1 × *Siglecg*‐R26^ki/ki^ mb1^cre^ mice, shown for individual mice over time A, BShown are percentages of B220^low^ CD5^+^ cells in the blood of individual mice over time in (A) comparison of TCL1 and TCL1 × Siglecg^−/−^ mice and in (B) TCL1 × *Siglecg*‐R26^ki/ki^ and TCL1 × *Siglecg*‐R26^ki/ki^ mb1^cre^ control mice. To distinguish different mice different symbols were used to represent individual mice that are connected by lines. *N* = 15 TCL1; *n* = 15 TCL1 × *Siglecg* ^−/−^; *n* = 22 TCL1 × *Siglecg*‐R26^ki/ki^; *n* = 17 TCL1 × *Siglecg*‐R26^ki/ki^ mb1^cre^.

Eμ‐TCL1 transgenic mice, here further referred to as TCL1 mice, were used as a mouse model for CLL. To investigate the influence of Siglec‐G deficiency on the development of CLL, TCL1 mice were crossed to Siglec‐G‐deficient (*Siglecg* ^−/−^) mice. Flow cytometric analyses of peripheral blood cells were performed at 4‐week intervals to monitor the development of B220^low^ CD5^+^ IgM^+^ CLL‐like cells, up until 60 weeks of age (Fig 1). Representative dot plots in Fig 1A show the population of B220^low^ CD5^+^ lymphocytes, which are B1a cells in wild type and *Siglecg* ^−/−^ mice and the expansion of CLL‐like cells in TCL1 and TCL1 × *Siglecg* ^−/−^ mice in the blood (Fig 1A). WT and *Siglecg* ^−/−^ controls revealed a low B1a cell population (B220^low^ CD5^+^) in WT (~ 2% of lymphocytes) and a higher B1a cell population in the blood of *Siglecg* ^−/−^ mice (~ 10% of lymphocytes). The size of these B1a cell populations in the two control mouse strains remained constant and did not increase until the age of 60 weeks (Fig 1B). TCL1 mice showed an increase of the population of CLL‐like cells (also characterized as B220^low^ CD5^+^) in the blood from the age of 40 weeks, reaching a percentage of about 50% of all blood lymphocytes at week 60. In contrast, the CLL‐like population in the blood of TCL1 × *Siglecg* ^−/−^ animals increased much earlier, already at the age of 20 weeks. This earlier increase of the CLL‐like population in TCL1 × *Siglecg* ^−/−^ mice was also evident from follow up of individual mice over the time course (Fig EV1A). From the age of 8 weeks up to the age of 40 weeks, the CLL‐like population in TCL1 × *Siglecg* ^−/−^ mice was significantly increased compared to TCL1 mice (Fig 1B). Of note, no single animal of the TCL1 × *Siglecg* ^−/−^ group survived until the age of 60 weeks.

**Figure EV2:**
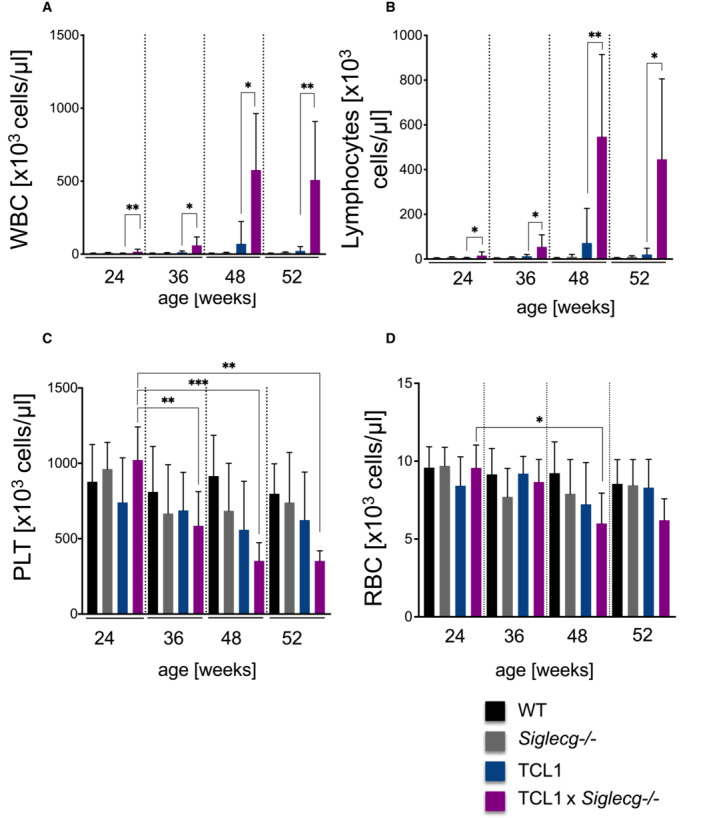
Earlier signs of leukocytosis and lymphocytosis in TCL1 × *Siglecg* ^−/−^ mice A–DFor hematological analysis of blood cells (A) the leukocyte count (B) the lymphocyte count (C) the platelet count and (D) the red blood cell count were determined with an Advia 120 hematology analysis machine. The mean values are shown with SD. Significant differences between groups were determined by one‐way ANOVA with Kruskal–Wallis and corrected for multiple comparison with Dunn's test, *P < 0.05, **P < 0.01, ***P < 0.001. *n* = 5–16 animals per time point and genotype, summarized from at least five independent experiments.

We extended our hematologic screening to determine whether the TCL1 animals develop blood leukocytosis and anemia in analogy to human CLL patients. As expected, the leukocyte numbers in the WT and *Siglecg* ^−/−^ controls remained consistently low. TCL1 mice only showed a moderate increase of total leukocyte and lymphocyte numbers over time (Fig EV2A and B). In contrast, we could observe a stronger progression of leukocytosis as well as lymphocytosis in TCL1 × *Siglecg* ^−/−^ mice with significantly increased cell numbers compared to TCL1 mice (Fig EV2A and B). While TCL1 mice did not show significant decreases of platelets or red blood cell numbers from 24 to 52 weeks of age, TCL1 × *Siglecg* ^−/−^ mice showed a significant reduction of platelet numbers in the same time interval (Fig EV2C). In addition, red blood cell numbers in TCL1 × *Siglecg* ^−/−^ mice seemed to decrease as the disease progressed (Fig EV2D).

### Earlier splenomegaly, earlier infiltrations of CLL‐like cells into various organs and lower survival of TCL1 × Siglecg^−/−^ mice

**Figure 2:**
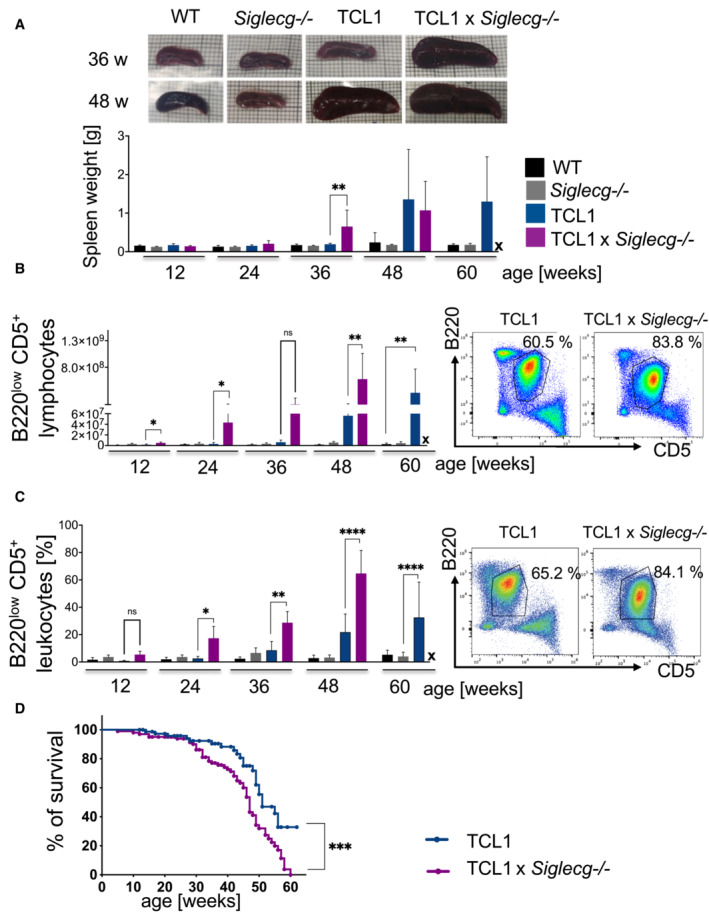
Earlier splenomegaly, earlier expansion, and infiltration of CLL‐like cells in spleen and liver and lower survival of TCL1 × *Siglecg* ^−/−^ mice Depicted are representative photographs of the spleen on graph paper of the different genotypes at 36 and 48 weeks of age. The spleen weight was determined and the mean values with ± SD are shown for every time point. Significant differences between groups were tested by one‐way ANOVA with Kruskal–Wallis test and corrected for multiple comparison with Dunn's test.The absolute cell numbers of B220^low^ CD5^+^ lymphocytes at different time points in the spleen are shown as mean values with ± SD. Significant differences between groups were tested by one‐way ANOVA with Kruskal–Wallis test and corrected for multiple comparison with Dunn's test. The different time points were tested separately. Alongside representative dot plots show selection and percentages of B220^low^ CD5^+^ cells in the spleen pre‐gated on single cells, living cells and lymphocytes.The percentage of B220^low^ CD5^+^ leukocytes in the liver at different time points is illustrated. Besides representative dot plots show the selection of B220^low^ CD5^+^ cells in the liver. Cells were pre‐gated on single cells, living cells and CD45^+^ leukocytes. Shown are mean values with ± SD. Significant differences between groups were tested by ordinary one‐way ANOVA with Šídák's *post‐hoc* test.The survival of TCL1 × *Siglecg* ^−/−^ mice compared to TCL1 mice is illustrated with the Kaplan–Meier survival curve (*n* = 81 for TCL1, *n* = 101 for TCL1 × *Siglecg* ^−/−^). The groups were compared for significant differences via log‐rank test. Data information: (A–C) Data are from at least 10 independent experiments for individual time points, comprising *n* = 5–12 animals per time point and genotype. X = no surviving TCL1 × *Siglecg* ^−/−^ animals at the age of 60 weeks. *P < 0.05, **P < 0.01, ****P < 0.0001. Source data are available online for this figure.

CLL progress is often accompanied by an enlargement of the spleen. We determined the development of splenomegaly in our TCL1 model at different time points. The spleen size or weight of TCL1 mice did not differ from those of WT animals up to the age of 36 weeks (Fig 2A). At the age of 48 weeks TCL1 mice showed significantly larger spleens with increased weight. In contrast, a significant enlargement of the spleen in TCL1 × *Siglecg* ^−/−^ animals was observed already at the age of 36 weeks (Fig 2A). At the age of 48 weeks both TCL1 and TCL1 × *Siglecg* ^−/−^ mice reach a comparable organ size. This correlated with CLL‐like cell expansions in the spleen of TCL1 mice from the age of 48 weeks onwards, while CLL‐like cell numbers increased much earlier in TCL1 × *Siglecg* ^−/−^ mice. In this strain, CLL‐like cell numbers were significantly higher than in TCL1 mice from the age of 12 weeks onwards (Fig 2B).

Infiltrations of CLL‐like cells into the liver of TCL1 mice could be observed from 48 weeks of age onwards (Fig 2C). TCL1 × *Siglecg* ^−/−^ mice showed significantly higher infiltration of CLL‐like cells into the liver from the age of 24 weeks onwards, when compared to TCL1 mice. Also, in other organs such as the peritoneal cavity or the bone marrow of TCL1 × *Siglecg* ^−/−^ mice, stronger expansions of the CLL‐like populations were detected (Appendix Fig S1A and B).

The survival of TCL1 and TCL1 × *Siglecg* ^−/−^ mice was monitored over time. Starting at the age of approximately 30 weeks a significant survival disadvantage of TCL1 × *Siglecg* ^−/−^ mice was evident (Fig 2D). Moreover, no TCL1 × *Siglecg* ^−/−^ animal reached the age of 60 weeks, contrasting with 30% of the TCL1 animals being still alive at this time point.

### TCL1 × Siglecg^−/−^ mice develop CLL‐like cells with a monoclonal Ig repertoire earlier than TCL1 mice

**Figure 3:**
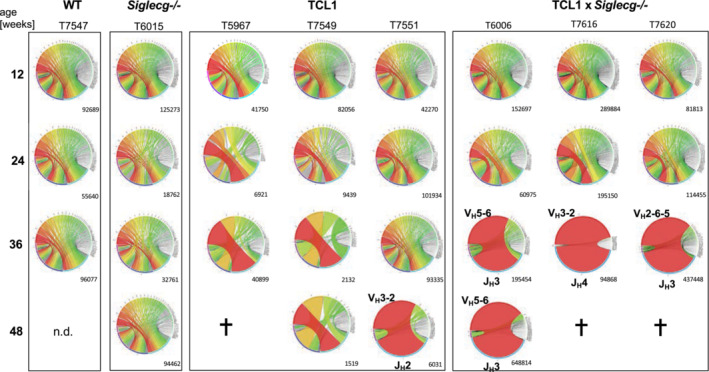
TCL1 × *Siglecg* ^−/−^ mice develop leukemic clones earlier than TCL1 controls Analysis of the IgV_H_ repertoire of CD19^+^ sorted blood cells by NGS. Circos plots depicting the frequencies of V_H_J_H_ usage from three individual mice for the different time points are shown. The number of productive sequences is indicated under the respective plot. The V_H_ family of prominent clones is highlighted. The cross indicates that the animals died before reaching the time point and therefore no analysis could be performed. n.d. indicates no data available. Data are from one experiment, comprising *n* = 3 mice per genotype.

In order to monitor tumor outgrowth, we performed a longitudinal analysis of the IgV_H_ repertoire of CLL‐like cells of TCL1 and TCL1 × *Siglecg* ^−/−^ mice. To achieve this, peripheral blood was collected at defined time points (12, 24, 36, and 48 weeks of age) to study individual mice over time. CD19^+^ B cells from the blood were sorted by flow cytometry, followed by RNA isolation and library preparation for Next Generation Sequencing (NGS). A high diversity of IgV_H_ sequences was found in the control animals (WT and *Siglecg* ^−/−^ *)* over the whole analysis period (Fig 3). Three individual TCL1 mice were analyzed from 12 to 48 weeks of age. Until the age of 36 weeks they showed a polyclonal or a slightly oligoclonal repertoire. At 48 weeks, one TCL1 mouse developed a monoclonal IgV_H_ repertoire with usage of a V_H_3‐2 element. One TCL1 mouse had an oligoclonal IgV_H_ repertoire, while one mouse died before the age of 48 weeks (Fig 3). In the three TCL1 × *Siglecg* ^−/−^animals a dominant monoclonal IgV_H_ repertoire was detected earlier in each mouse tested (involving V_H_2, V_H_3, and V_H_5 segments, respectively), starting already at the age of 36 weeks. One mouse kept this clone until 48 weeks, the other two mice died before reaching the age of 48 weeks. These results indicate that TCL1 × *Siglecg* ^−/−^ animals do not only show an earlier lymphoproliferation of their CLL‐like cells, but also develop leukemic monoclonal expansions earlier than TCL1 mice. All monoclonal IgV_H_ sequences showed a low rate (0–3.7%) of IgV_H_ mutations (Appendix Table S1).

CLL‐like cells frequently express a BCR repertoire with V_H_ segments that are typical for B1a cells, such as V_H_11 and V_H_12, binding characteristic autoantigens (Yan *et al*, 2006). We performed staining of CLL‐like cells with phosphatidylcholine‐ (PtC) containing liposomes and phosphorylcholine‐ (PC)‐BSA that are bound by BCRs often carrying V_H_11 and V_H_12 elements. This analysis revealed a significantly reduced binding of these antigens in TCL1 × *Siglecg* ^−/−^ mice in comparison to TCL1 littermates for most time points (Appendix Fig S2A and B). This suggests an overall different BCR repertoire of CLL‐like cells of TCL1 × *Siglecg* ^−/−^ mice, when compared to TCL1 mice. A similar shift in the BCR repertoire was detected previously in normal B1a cells of *Siglecg* ^−/−^ mice (Jellusova *et al*, 2010).

### Siglec‐G overexpression on B cells leads to a strong suppression of CLL‐like cells in the blood

**Figure 4:**
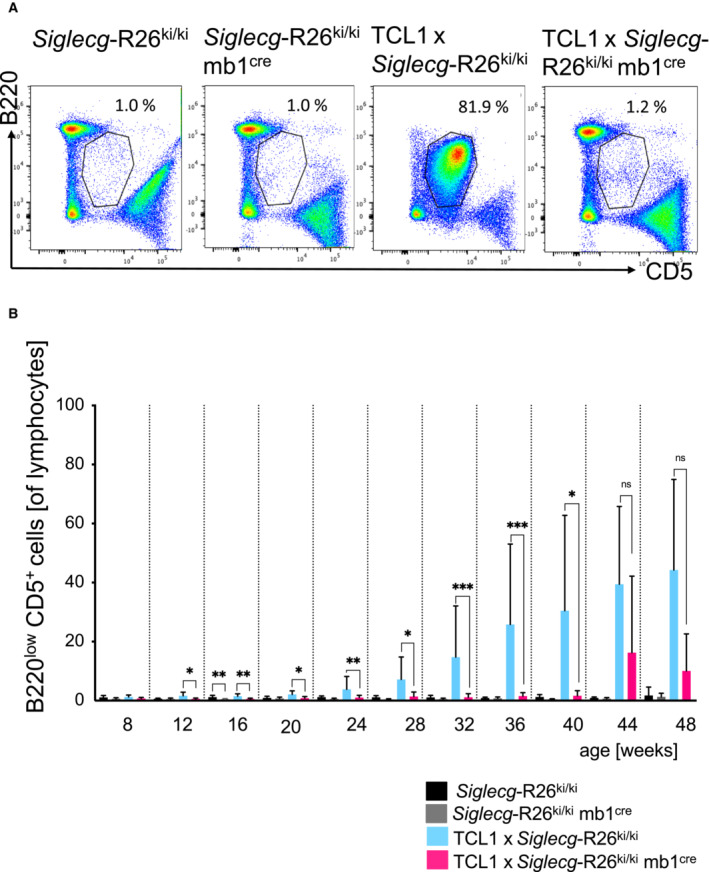
Overexpressed Siglec‐G prevents development of CLL‐like cells in the blood of TCL1 mice almost completely To monitor the progression of the CLL‐like population, blood was collected from the animals every 4 weeks up to an age of 48 weeks and analyzed by flow cytometry. Genotypes of the mice are: controls (*Siglecg*‐R26^ki/ki^), Siglec‐G overexpressing mice (*Siglecg*‐R26^ki/ki^ mb1^cre^), transgenic TCL1 controls (TCL1 × *Siglecg*‐R26^ki/ki^) and transgenic TCL1 Siglec‐G overexpressing mice (TCL1 × *Siglecg*‐R26^ki/ki^ mb1^cre^). Representative FACS plots show examples of stainings of B220^low^ CD5^+^ B cells. (CLL‐like cells) and the selected gate for (B).The diagram displays the percentage of B220^low^ CD5^+^ cells in respect to lymphocytes over a time period of 48 weeks. Shown are mean values with ± SD. Significant differences between groups were determined by one‐way ANOVA with Kruskal–Wallis test (no normal distribution) and corrected for multiple comparison with Dunn's test. All time points were tested separately. *P < 0.05, **P < 0.01, ***P < 0.001. Summarized data from more than 20 independent experiments with *n* = 5–20 animals per time point and genotype are shown. Source data are available online for this figure.

**Figure EV3:**
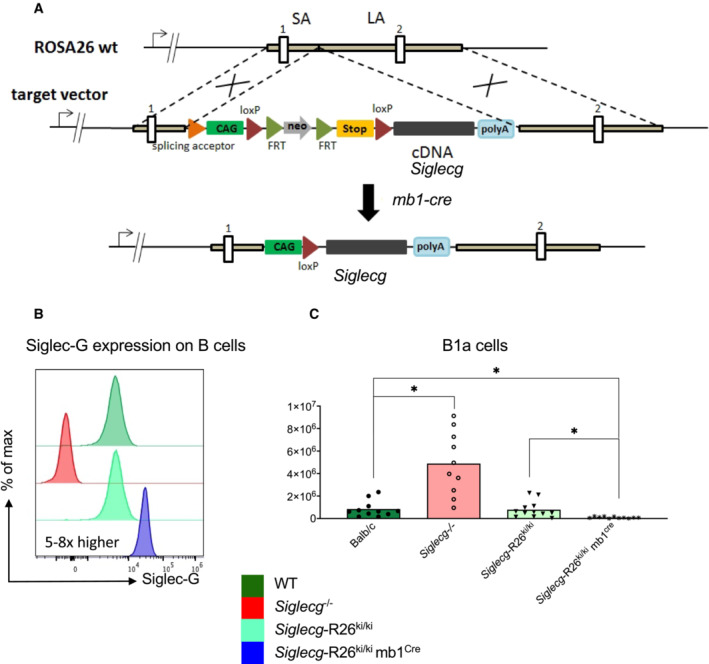
Generation of Siglec‐G overexpressing mice For the generation of Siglec‐G overexpressing mice the cDNA of Siglec‐G was cloned into the target vector, between the short (SA) and the long arm (LA) of the ROSA26 (R26) locus. Overexpression was facilitated by the CAG enhancer cassette, which contains the chicken ß‐actin promoter and the early enhancer element of cytomegalovirus. A transcriptional stop cassette flanked by two loxP sites ensured B cell‐specific expression by mating with the mb1cre mouse strain. The cre recombinase specifically removes the stop cassette so that the enhancer cassette is brought in front of the cDNA and transcription of the Siglec‐G cDNA occurs.Siglecg‐R26^ki/ki^ mb1^cre^ mice showed a five to eightfold overexpression of Siglec‐G on the surface of mature B cells in the spleen and peritoneal cavity, respectively. The histograms represent the expression of Siglec‐G on conventional B2 cells of the spleen.In the peritoneal cavity a reduction of B1a cells was observed in Siglecg‐R26^ki/ki^ mb1^cre^ mice in contrast to the enlargement of this population in Siglecg^−/−^ mice. Shown are the mean values of the absolute cell counts. Cells were pre‐gated for single, living lymphocytes subsequently, B1a cells were identified as B220^low^ and CD5^+^. Significant differences between groups were determined with Kruskal–Wallis and corrected for multiple comparison with Dunn's test, *P < 0.05. *n* = 6–14 animals per genotype; every dot represents a mouse. Data are summarized from 5 independent experiments.

**Figure EV4:**
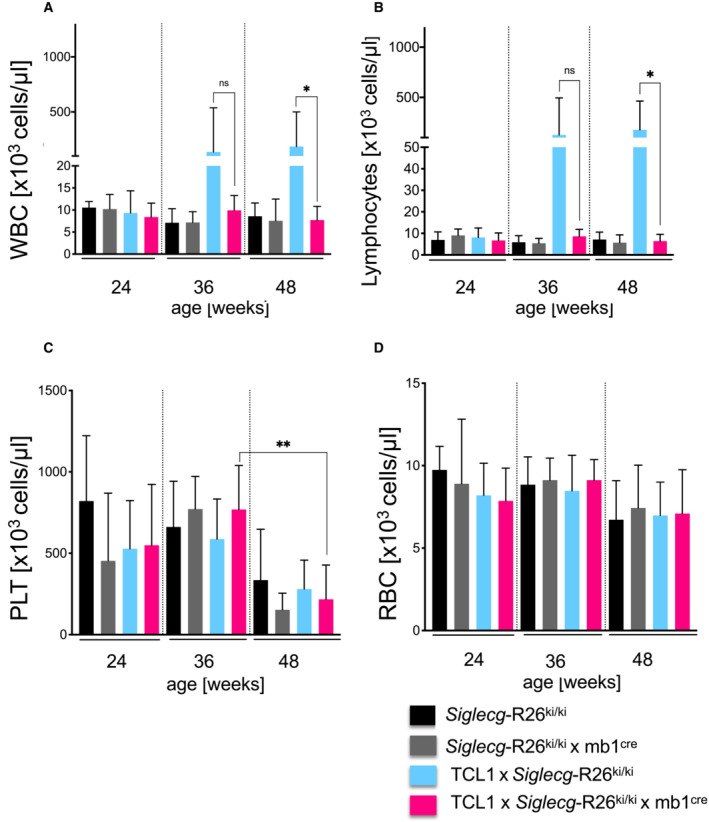
No leukocytosis or lymphocytosis in TCL1 × *Siglecg*‐R26^ki/ki^ × mb1^cre^ mice A–DFor hematological analysis of blood cells (A) the leukocyte count (B) the lymphocyte count (C) the platelet count and (D) the red blood cell count were determined with an Advia 120 hematology analysis machine. The mean values are shown with ± SD. Significant differences between groups were determined by one‐way ANOVA with Kruskal–Wallis and corrected for multiple comparison with Dunn's test, *P < 0.05, **P < 0.01. *n* = 5 animals per time point and genotype, summarized from at least 5 independent experiments.

Since we found that Siglec‐G deficiency led to an earlier development of the CLL‐like disease in TCL‐1 mice, we aimed to test whether overexpression of this inhibitory receptor might suppress the disease or lead to a favorable outcome. To test this hypothesis, Siglec‐G overexpressing mice (*Siglecg*‐R26^ki/ki^) were generated by a *knockin* of *Siglecg* cDNA into the Rosa26 locus (Fig EV3A). The Siglec‐G cDNA is under the control of a CAG promoter followed by a floxed transcriptional stop cassette, which is removed by crossing with mb1‐cre mice, resulting in B cell‐specific overexpression. The characterization of Siglec‐G overexpressing mice (*Siglecg*‐R26^ki/ki^ mb1^cre^) revealed a B‐cell specific overexpression of Siglec‐G on the surface of B‐lineage cells, starting at the pro/pre B cell stage in bone marrow and continuing on all peripheral B cells. Mature B cells in the spleen showed a fivefold overexpression, while the B1a cell population in the peritoneum showed an approximately eightfold overexpression of Siglec‐G (Fig EV3B). In contrast to the enlargement of the B1a cell population in *Siglecg* ^−/−^ mice, a significant reduction of this population could be observed upon overexpression of *Siglecg* (Fig EV3C, about 8‐fold reduction of B1a cell numbers). Siglec‐G overexpressing mice had a normal B‐cell development in the bone marrow and showed overall normal splenic B‐cell subpopulations (Appendix Fig S3A and B). In order to test the influence of overexpressed Siglec‐G on the CLL disease, *Siglecg*‐R26^ki/ki^ mb1^cre^ mice were crossed with TCL1 mice. To monitor the progression of the leukemia‐like population in the blood by flow cytometric analysis, blood was drawn in 4‐week intervals until the age of 48 weeks. The representative dot plots in Fig 4A show that the CLL‐like population was significantly reduced in TCL1 × *Siglecg*‐R26^ki/ki^ mb1^cre^ mice (Siglec‐G overexpressing) compared to TCL1 × *Siglecg*‐R26^ki/ki^ transgenic control mice. This effect can also be observed over time. TCL1 × *Siglecg*‐R26^ki/ki^ mice showed a constantly increasing CLL‐like population starting at the age of about 28 weeks, which expanded steadily over time and reached its peak of about 40–50% of all blood lymphocytes at the age of 48 weeks (Fig 4B). This is consistent with the results of TCL1 mice of the previously analyzed cohort (Fig 1B). In contrast, the CLL‐like population in TCL1 × *Siglecg*‐R26^ki/ki^ mb1^cre^ mice remained at a consistently low level (≤ 1%), which is comparable to the non‐transgenic controls, over time and was always significantly lower than TCL1 × *Siglecg*‐R26^ki/ki^ mice (Fig 4B). However, starting at 44 weeks of age, a rise in this population was evident in TCL1 × *Siglecg*‐R26^ki/ki^ mb1^cre^ mice (to about 10% of blood lymphocytes), even though it was still substantially reduced compared to TCL1 × *Siglecg*‐R26^ki/ki^ mice. The analysis of individual mice over time showed that only some mice of the TCL1 × *Siglecg*‐R26^ki/ki^ mb1^cre^ group showed an increased CLL‐like population after 44 weeks of age, while most mice of this group remained very low in this population (Fig EV1B). The age matched non‐transgenic controls stayed at a constant low level over the course of 48 weeks, while *Siglecg*‐R26^ki/ki^ mb1^cre^ showed a tendency to a smaller B1a population (Fig 4B). Corresponding to the very delayed and only modest increase of the CLL‐like population in the blood of TCL1 × *Siglecg*‐R26^ki/ki^ mb1^cre^ animals, no signs of leukocytosis and lymphocytosis were observed in the blood cell analysis of these mice (Fig EV4A and B). In contrast, TCL1 × *Siglecg*‐R26^ki/ki^ mice revealed significantly elevated numbers of leukocytes and lymphocytes beginning at the age of 36 weeks. The platelet and red blood cell numbers did not show any gross changes. Lower numbers of platelets were observed for all four genotypes at 48 weeks, reaching significance for the TCL1 × *Siglecg*‐R26^ki/ki^ mb1^cre^ mice, for unknown reasons (Fig EV4C and D).

### Siglec‐G overexpression prevents splenomegaly, infiltrations of CLL‐like cells into various organs and significantly prolongs survival of TCL1 mice

**Figure 5:**
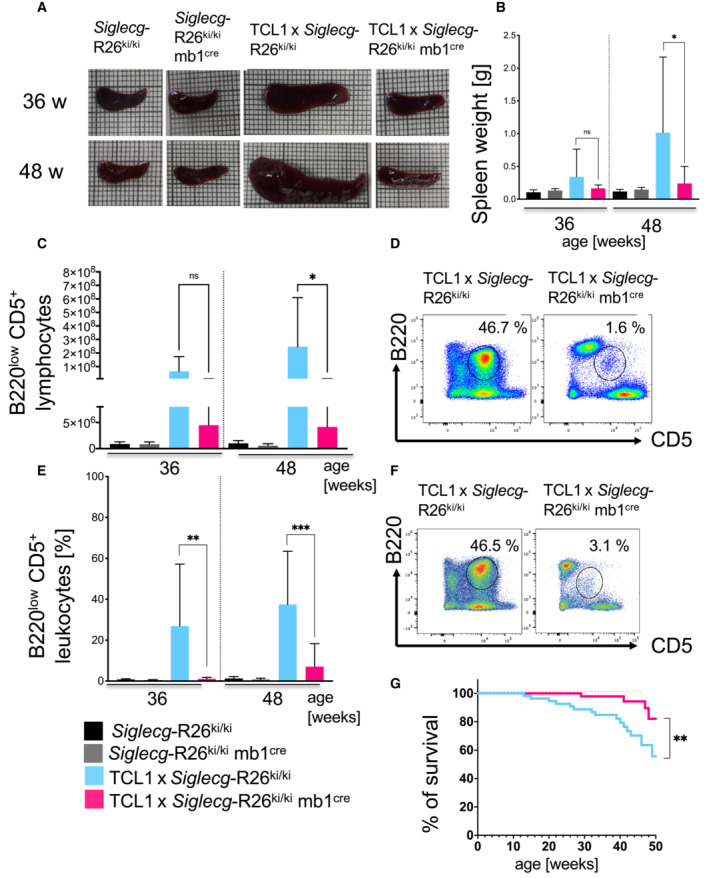
No splenomegaly, hardly any expansion of CLL‐like cells in spleen and liver and better survival of Siglec‐G overexpressing TCL1 mice Depicted are representative photographs of the spleen on graph paper of the different genotypes at 36 and 48 weeks of age.The spleen weight in g was determined and the mean values with ± SD are shown for every time point. Significant differences between groups were tested by one‐way ANOVA with Kruskal–Wallis test and corrected for multiple comparison with Dunn's test. *n* = 8–15 animals per time point and genotype.Absolute cell numbers of B220^low^ CD5^+^ lymphocytes at 36 and 48 weeks of age in the spleen are shown as mean values with ± SD. Significant differences between groups were tested by one‐way ANOVA with Kruskal–Wallis test and corrected for multiple comparison with Dunn's test. The different time points were tested separately. *n* = 8–13 animals per time point and genotype.Representative dot plots show selection and percentages of B220^low^ CD5^+^ B cells in the spleen pre‐gated on single cells, living cells and lymphocytes.The percentage of B220^low^ CD5^+^ lymphocytes in the liver at 36 and 48 weeks of age is illustrated. Shown are mean values with ± SD. Significant differences between groups were tested by ordinary one‐way ANOVA with Šídák's *post‐hoc* test. *n* = 7–14 animals per time point and genotype.Representative dot plots for the selection of B220^low^ CD5^+^ cells in the liver. Cells were pre‐gated on single cells, living cells and CD45^+^ leukocytes.The differences in survival are shown as Kaplan–Meier plots and log‐rank test *n* = 59 for TCL1 × *Siglecg*‐R26^ki/ki^, *n* = 57 for TCL1 × *Siglecg*‐R26^ki/ki^ mb1^cre^. Data information: (B), (C), (E). Data are from at least 10 independent experiments for individual time points. *P < 0.05, **P < 0.01, ***P < 0.001. *n* = 48 for TCL1 × *Siglecg*‐R26^ki/ki^, *n* = 52 for TCL1 × *Siglecg*‐R26^ki/ki^ mb1^cre^. Source data are available online for this figure.

In TCL1 × *Siglecg* ^−/−^ animals an earlier occurrence of splenomegaly, due to earlier infiltrations of CLL‐like cells, had been detected (Fig 2A and B). As a result of the delayed increase of the CLL‐like population in the blood of TCL1 × *Siglecg*‐R26^ki/ki^ mb1^cre^ mice (Fig 4B), we hypothesized that splenomegaly would occur later. As expected, TCL1 × *Siglecg*‐R26^ki/ki^ animals showed a significant enlargement of the spleen starting at the age of 36 weeks, which was also confirmed regarding the organ weight (~ 0.7 g at 48 weeks). In contrast, no enlargement of the spleen was found in TCL1 × *Siglecg*‐R26^ki/ki^ mb1^cre^ mice, as organ size and weight were comparable to the non‐transgenic controls (≤ 0.15 g) (Fig 5A and B). This finding is supported by the fact that less CLL‐like cells expanded in the spleen of TCL1 × *Siglecg*‐R26^ki/ki^ mb1^cre^ mice, whereas in TCL1 × *Siglecg*‐R26^ki/ki^ mice a substantially enlarged CLL‐like population was detected (Fig 5C and D). This trend was also present in other lymphoid organs such as bone marrow and peritoneal cavity. There was hardly any infiltration of CLL‐like cells observed in the liver of TCL1 × *Siglecg*‐R26^ki/ki^ mb1^cre^ mice, whereas there was a high infiltration into the liver of TCL1 × *Siglecg*‐R26^ki/ki^ mice (Fig 5E and F). In terms of survival, Siglec‐G overexpression resulted in a significant survival advantage, as observed for TCL1 × *Siglecg*‐R26^ki/ki^ mb1^cre^ mice, when compared to TCL1 × *Siglecg*‐R26^ki/ki^ mice (Fig 5G).

### Siglec‐G overexpressing TCL1 mice do not develop CLL‐like cells with a monoclonal Ig repertoire

**Figure 6:**
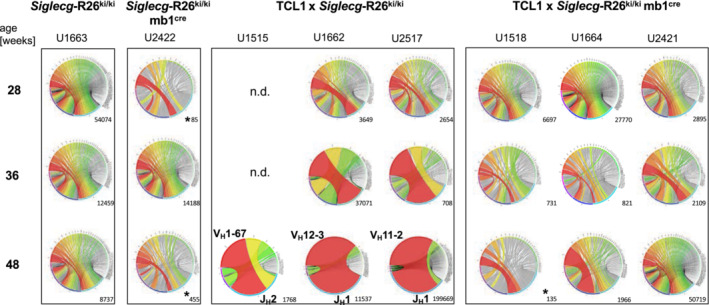
No indication of leukemic clone development in Siglec‐G overexpressing TCL1 mice Analysis of the IgV_H_ repertoire of CD19^+^ sorted blood cells by NGS. Circos plots depicting the frequencies of V_H_J_H_ usage from three individual mice for the different time points are shown. The number of productive sequences is indicated under the respective plot. When less than 500 productive sequences were obtained, these were marked with an asterisk. The V_H_ family of prominent clones is highlighted. n.d. indicates no data available. Data are from one experiment, comprising *n* = 3 mice per genotype.

Again, a longitudinal study was performed to detect the development of leukemic clones. For analysis of the IgV_H_ repertoire of CLL‐like cells, blood of individual mice was collected at defined three time points (28, 36, and 48 weeks of age). This RNA sequence analysis was again performed with sorted CD19^+^ cells from the blood. A high diversity of IgV_H_ sequences was found in the non‐transgenic control animals (*Siglecg*‐R26^ki/ki^ and *Siglecg*‐R26^ki/ki^ mb1^cre^) at all analysis time points (Fig 6). While three TCL1 × *Siglecg*‐R26^ki/ki^ mice all showed a clonal IgV_H_ sequence at 48 weeks, including rearrangements of the V_H_1, V_H_11 and V_H_12 families, respectively, there was no clonal IgV_H_ sequence detected in three TCL1 × *Siglecg*‐R26^ki/ki^ mb1^cre^ mice, until the age of 48 weeks (Fig 6). This shows that Siglec‐G overexpression suppresses the development of leukemic clones within the CLL‐like population.

### Siglec‐G expression affects anti‐BCR induced Ca^2+^ mobilization and proximal intracellular signaling pathways in CLL‐like cells

**Figure 7:**
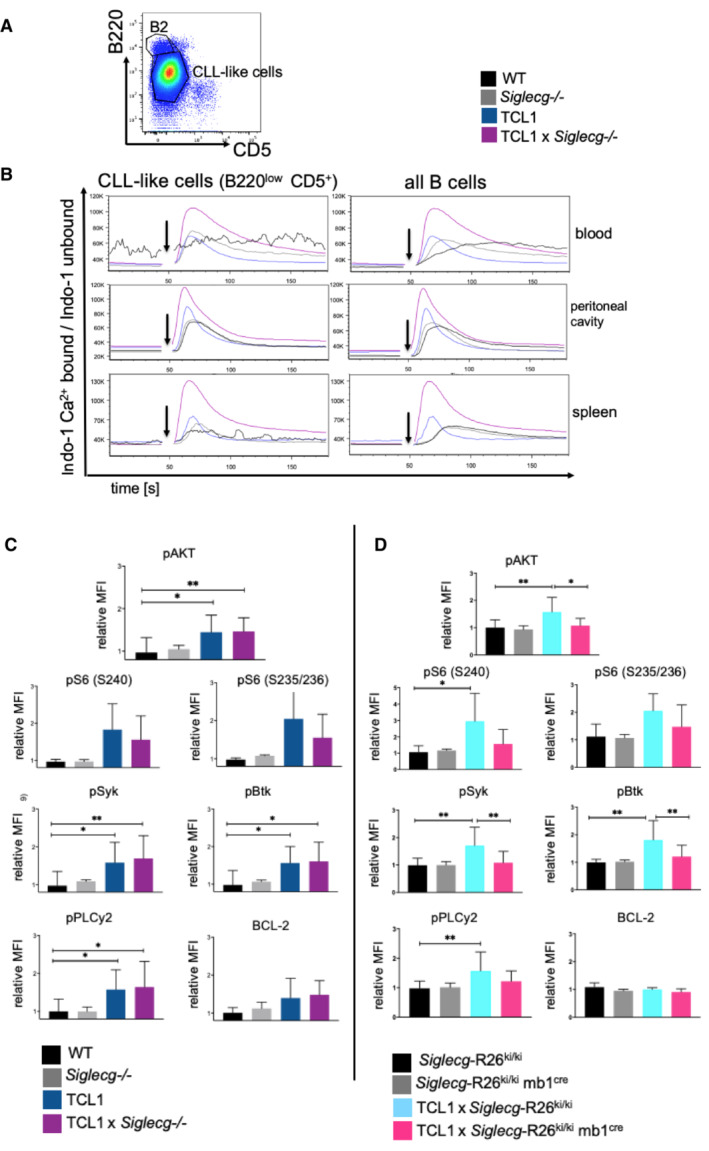
Higher BCR‐induced calcium response in CLL‐like cells of TCL1 × *Siglecg* ^−/−^ mice and increased intracellular signaling responses in TCL1 and TCL1 × *Siglecg* ^−/−^ mice, but not in Siglec‐G overexpressing TCL1 mice Shown is a representative dot plot for the selection strategy for CLL‐like B cells from the spleen of TCL1 × Siglecg^−/−^ mice. For the analysis of all B cells (B220+ and B220^low^ cells), conventional B2 cells plus the B220^low^ CD5^+^ population was used. Cells were pre‐gated for single cells and lymphocytes.To measure calcium influx cells were loaded with Indo‐1. Shown is the mean calcium concentration as ratio of bound to unbound Indo‐1 versus time in s. First the basal level was monitored for 50 s in Krebs–Ringer solution. Subsequently, the BCR was stimulated with 13 μg/ml anti‐IgM F(ab)_2_ (shown by the black arrow). Shown is one representative result of four independent experiments. Mice were between 36 and 48 weeks of age. To measure intracellular signaling splenic cells were pre‐gated on single, living cells and lymphocytes, then B220^+^ cells, including B220^low^ CD5^+^ lymphocytes were selected.TCL1 and TCL1 × *Siglecg* ^−/−^ B cells were analyzed by intracellular staining with phopho‐specific antibodies (or total protein detecting antibody Bcl2).TCL1 × *Siglecg*‐R26^ki/ki^ or TCL1 × *Siglecg*‐R26^ki/ki^ mb1‐cre B cells were analyzed by intracellular staining with phopho‐specific antibodies (or total protein detecting antibody Bcl2). Data information: Shown are geometric mean values normalized to the WT with ± SD. Significant differences between groups were tested either by ordinary one‐way ANOVA with Šídák's *post‐hoc* test if there was a normal distribution, or by one‐way ANOVA with Kruskal–Wallis test and corrected for multiple comparison with Dunn's test if there was no overall normal distribution. *P < 0.05, **P < 0.01. Data are from at least five independent experiments, summarized for *n* = 7–11 animals. Mice were between 36 and 48 weeks of age. Source data are available online for this figure.

BCR signaling plays a crucial role in the pathogenicity of CLL and is important for the maintenance of CLL cells (Burger & Chiorazzi, 2013; Schmid *et al*, 2022). In order to examine the influence of deficiency or overexpression of Siglec‐G on signaling pathways, CLL cells of both of our genetically modified TCL1 mouse models were analyzed for BCR‐induced Ca^2+^ responses and intracellular signaling pathways. First, Ca^2+^ responses of CLL‐like cells of TCL1 × *Siglecg* ^−/−^ and TCL1 mice of blood, spleen, and peritoneum were measured after stimulation with different concentrations of anti‐IgM F(ab)_2_. For the experiment CLL‐like cells (B220^low^CD5^+^), as well as all B cells, which means in this case conventional B2 cells combined with the B220^low^ CD5^+^ population, were selected as indicated in Fig 7A. The observed calcium mobilization, shown as the ratio of calcium‐bound Indo‐1 to unbound Indo‐1, after BCR stimulation with different concentrations of anti‐IgM F(ab)_2_ was significantly increased in both CLL‐like cells or in all B cells of TCL1 × *Siglecg* ^−/−^ mice compared to cells of TCL1 mice in all analyzed organs (Fig 7B). Furthermore, a higher calcium mobilization in TCL1 cells was observed in comparison to WT and *Siglecg* ^−/−^ cells. The baseline before stimulation, which corresponds to the cytosolic calcium level in resting cells, was comparable within the different cohorts. Attempts to measure BCR‐induced Ca^2+^ responses in Siglec‐G overexpressing mice were not successful due to the very low number of B220^low^CD5^+^ cells in *Siglecg*‐R26^ki/ki^ mb1^cre^ and TCL1 × *Siglecg*‐R26^ki/ki^ mb1^cre^ mice and due to inconsistent results when gating on total B cells.

We further elucidated downstream signaling pathways of the BCR to identify potential changes explaining the strong influence of Siglec‐G on CLL development. For this, the phosphorylation status of essential signaling molecules in B cells/CLL‐like cells was analyzed by flow cytometry. TCL1 is known to strongly activate the AKT signaling pathway and this is assumed to lead to the development of CLL in the Eμ‐TCL1 mouse model (Teitell, 2005). Thus, phosphorylation of AKT was determined and found to be increased in B cells of both TCL1 and TCL1 × *Siglecg* ^−/−^ mice in comparison to the WT (Fig 7C). However, no clear difference could be found between the two cohorts. Also, S6 phosphorylation was increased in both TCL1 and TCL1 × *Siglecg* ^−/−^ mice. Furthermore, phosphorylation of proximal signaling proteins, such as BTK, SYK/ZAP70 and PLCγ2 was increased in both types of mice when compared with wild type. In addition, BCL‐2, an anti‐apoptotic protein which is found to be overexpressed in CLL, was elevated in both TCL1 and TCL1 × *Siglecg* ^−/−^ mice, compared to WT mice (Fig 7C). Thus, although Siglec‐G deficiency led to clearly increased Ca^2+^ signaling in CLL‐like cells, the basic activation of several intracellular signaling proteins was not further enhanced.

While CLL‐like cells of TCL1 × *Siglecg*‐R26^ki/ki^ mice showed a similar increase of AKT, S6 phosphorylation and also of phosphorylation of the proximal signaling proteins Syk, BTK and PLCγ2 as in the previous TCL1 control cohort, this increased phosphorylation of all of these proteins was not observed in Siglec‐G overexpressing TCL1 cells (Fig 7D). Cells of these mice had similar protein phosphorylation levels as non‐transgenic WT B cells. Interestingly, no difference in expression of BCL‐2 could be observed in cells from TCL1 × *Siglecg*‐R26^ki/ki^ and TCL1 × *Siglecg*‐R26^ki/ki^ × mb1^cre^ mice. Thus, the typical activation of several signaling pathways in TCL1 transgenic CLL‐like cells could not be found when Siglec‐G was over‐expressed. Although the percentage of CLL‐like cells analyzed in Fig 7D was on average not different between TCL1 × *Siglecg*‐R26^ki/ki^ and TCL1 × *Siglecg*‐R26^ki/ki^ × mb1^cre^ mice, the latter had more widespread CLL‐like numbers (Appendix Fig S4). Therefore, their normal phopho‐protein levels maybe also due to the higher percentage of normal B cells.

### Siglec‐10 downmodulation from the surface of CLL cells in humans

**Figure 8:**
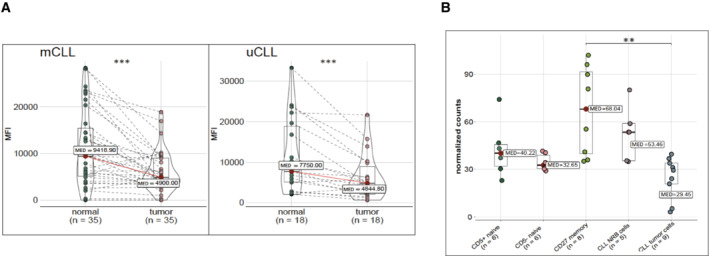
Downmodulation of human Siglec‐10 surface expression on CLL cells compared to normal residual B cells Peripheral blood B cells of CLL patients are pre‐gated on single, living B lymphocytes (CD19^+^). The mean fluorescence intensity (MFI) of surface Siglec‐10 is given from 35 IgV‐mutated (left) and 18 IgV‐unmutated CLL cases (right), always including tumor cells (CD20^low^CD5^high^) and normal residual B cells (CD20^high^CD5^−^) as paired samples.The transcript expression (normalized counts from bulk mRNA sequencing) of SIGLEC10 is given from five healthy donors, discriminating naïve CD5^−^ (5 donors), mature CD5^+^ (86 donors), and CD27^+^ memory B‐cell (8 donors) subsets, from CLL tumor cells (*n* = 9) and from five paired normal residual B (NRB) cells (*n* = 5). Data information: Wilcoxon rank sum test, **P < 0.01, ***P < 0.001. Median as central band, box encompassed from first to third quartile, whiskers are the smallest or largest value no further than 1.5 * IQR (range from first to third quartiles) from the hinge. Source data are available online for this figure.

**Figure EV5:**
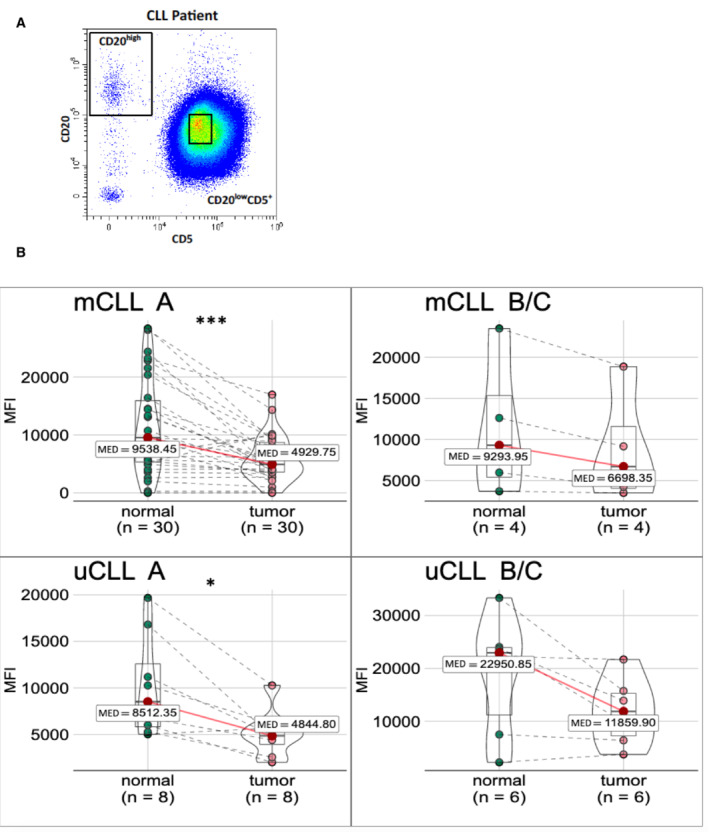
Downmodulation of human Siglec‐10 surface expression on CLL cells compared to normal residual B cells Peripheral blood B cells, pre‐gated on single, living B lymphocytes (CD19^+^). One representative plot for the gating of CLL cells (CD20^low^CD5^high^) and normal residual (NRB) cells (CD20^high^CD5^−^) is given.Data as in Fig 8A, separated by IgV‐mutation status (mCLL and uCLL, top and bottom row, respectively) and Binet A versus Binet B or C stage of the patient (left versus right column). Data information: Wilcoxon signed‐rank test, *P < 0.05, ***P < 0.001. Samples are paired biological replicates. Median as central band, box encompassed from first to third quartile, whiskers are the smallest or largest value no further than 1.5 * IQR (range from first to third quartiles) from the hinge.

Based on the observed influence of Siglec‐G expression levels on the development of CLL‐like cells in the mouse we also studied Siglec‐10, the human orthologue to Siglec‐G, on human primary CLL cells from patient blood samples. Thus, we determined the Siglec‐10 surface expression on CLL cells (gated as CD20^low^ CD5^+^) and the corresponding normal residual B cells (gated as CD20^high^ CD5^−^, Fig EV5A) from patient blood samples in a cohort of 73 CLL cases. Among 35 cases with confirmed IgV‐mutated, and 18 cases with IgV‐unmutated status, we observed a high variation in the Siglec‐10 surface expression level, but overall observed a significant downmodulation of Siglec‐10 on the surface of CLL cells compared to the corresponding normal residual B cell population, independent of the IgV‐mutation status of the tumor (Fig 8A), or BINET stage of the patient (Fig EV5B). The fold‐change of Siglec‐10 downmodulation between normal and tumor cells was on average higher among CLL cases with unfavorable prognosis (uCLL vs. mCLL) (Appendix Fig S5A), however, did not reach statistical significance. Anticipating that the tumor environment in CLL patients may affect *SIGLEC10* expression, we also included normal naïve CD5^−^ B cells, naïve CD5^+^ B cells, and CD27^+^ memory B cells from 5 age‐matched healthy donors. This showed that normal B cell subsets express similar levels of surface Siglec‐10 (memory B cells more than naive B cells), and moreover, these surface expression levels on average exceed those of CLL tumor cells (Appendix Fig S5B). These observations were also reflected by *SIGLEC10* mRNA expression levels among B cell subsets from healthy adults (*n* = 5 each) and tumor and normal residual B cells from 9 CLL patients, as determined by RNAseq (Fig 8B). There was a trend of lower *SIGLEC10* mRNA expression in CLL cells, compared to their normal residual B cells. In total, these data show a downmodulation of the inhibitory Siglec‐10 protein from the surface of CLL cells in humans, and this effect is tendentiously stronger among IgV‐unmutated cases.

## Discussion

In this study we show in the EμTCL1 mouse model that Siglec‐G plays a crucial role for the development of CLL. The loss of this inhibitory receptor leads to an earlier onset and a more severe course of the disease. This is reflected by an earlier and stronger accumulation of the leukemic cells in the blood, as well as earlier and elevated expansion and infiltration of CLL‐like cells into various organs such as spleen, liver, bone marrow, and peritoneal cavity. Finally, the more severe disease progression is indicated by a survival disadvantage of the TCL1 × *Siglecg* ^−/−^ animals. Since Siglec‐G is a negative regulator of BCR signaling in normal CD5^+^ B1a cells (Hoffmann *et al*, 2007) and since BCR signaling is crucial for CLL development and maintenance (Burger & Chiorazzi, 2013; Schmid *et al*, 2022), these results were not unexpected. *Siglecg* ^−/−^ B1a cells show much stronger BCR‐induced Ca^2+^ signaling (Hoffmann *et al*, 2007) and this enhanced Ca^2+^ signaling was also found in CLL‐like cells in this study. The dependence of CLL on BCR signaling is not due to BCR‐activating mutations in crucial signaling molecules as shown for diffuse B cell lymphoma (DLBCL) (Schmitz *et al*, 2018), but due to the ability of some CLL BCRs to cluster by binding to each other and in this way autonomously activating BCR signaling (Duhren‐von Minden *et al*, 2012; Minici *et al*, 2017). The induction of BCR signals by low‐affinity autoantigens also plays a role in CLL development (Iacovelli *et al*, 2015). The successful use of BTK inhibitors as therapy for CLL further demonstrates the crucial role of BCR signaling in CLL, as BTK is a crucial proximal kinase in this signaling pathway (Burger & Chiorazzi, 2013). Accordingly, either genetic deletion of the BTK gene or inhibition of BTK by ibrutinib in the Eμ‐TCL1 mouse model significantly delays the onset of CLL (Woyach *et al*, 2014). A recent study also showed that CLL cells cannot survive after inducible deletion of the BCR from their surface (Schmid *et al*, 2022). This finding indicates the importance of the BCR not only for induction, but also for maintenance of CLL. Another CLL mouse model, expressing a SV40 large T insertion in the IgH locus, also showed an earlier and stronger CLL‐like disease, when crossed to the *Siglecg* ^−/−^ background (Pal Singh *et al*, 2018), supporting our findings with EμTCL1 mice in this study.

We consider our results of overexpression of Siglec‐G in the TCL1 model on the disease progression as quite remarkable. Just about fivefold overexpression of this inhibitory receptor on the cellular surface resulted in hardly any detectable CLL‐like population and strongly delayed lymphoproliferation in the blood, which was also confirmed by the absence of leukocytosis in these mice. In the same way, infiltrations of CLL cells in all organs were substantially reduced, no leukemic clones were detected by our analysis and overall these mice demonstrated a survival advantage, when compared to control TCL1 transgenic mice. Overexpression of Siglec‐G prevented the over‐activation of the PI3‐K pathway, as well as the activation of proximal BCR signaling, which are both pathways upregulated in CLL cells. However, this normal signaling phenotype may also be a consequence of the fact that CLL‐like cells did hardly develop in Siglec‐G overexpressing TCL1 mice. Overall, this suggests that agonistic targeting of the inhibitory receptor Siglec‐G could lead to suppression of the CLL disease.

Besides its direct influence as inhibitory receptor on BCR signaling, Siglec‐G also regulates the size of the CD5^+^ B1a cell population. *Siglecg* ^−/−^ mice have an up to 10‐fold increased B1a cell population (Hoffmann *et al*, 2007), while this population is about eightfold decreased in overexpressing *Siglecg*‐R26^ki/ki^ mb1^cre^ mice. The cellular origin of human CLL cells is either from CD5^+^ naïve or CD5^+^ CD27^+^ memory B cells, as indicated by respective very similar transcriptional profiles (Seifert *et al*, 2012). Murine CD5^+^ B1a cells have some similar characteristics to human CD5^+^ CLL cells, for example, by expressing a restricted IgV_H_ repertoire with a preferred usage of V_H_11 and V_H_12 families, including specificities for autoantigens and microbial antigens (Herve *et al*, 2005; Baumgarth, 2011). Similarly, over‐usage of the V_H_11, V_H_12, and V_H_4 gene segments has been demonstrated in TCL1 animals. Each of these three V_H_ families was used in about 10% of leukemic clones of TCL1 transgenic mice, while the rest of leukemic clones used other V_H_ families (Yan *et al*, 2006). Several lines of evidence suggest that a restricted BCR repertoire and specific antigen selection drive disease progression (Chiorazzi & Ferrarini, 2003). For instance, the IgV_H_ mutation status determines the course of disease and survival predictions (Fais *et al*, 1998). In addition, BCRs with remarkable structural similarity could be identified between different patients (Tobin *et al*, 2003). Therefore, the changed size of the CD5^+^ B cell precursor population in Siglec‐G‐deficient or ‐overexpressing mice could influence the development of the CLL‐like cells in the TCL1 mouse model. Within the group of control TCL1 mice leukemic clones using V_H_11 in one mouse and V_H_12 in a second mouse, among 4 mice in total were found. V_H_11 and V_H_12 segments did not occur in three *Siglecg* ^−/−^ TCL1 mice. Certainly, the mouse numbers of three or four mice per group are too low to quantify V_H_ usage in these two genotypes. The IgV_H_ analysis was done as a longitudinal study to monitor the time point of developing leukemic clones, therefore not so many mice per group were analyzed. However, staining with the typical antigens that are often recognized by V_H_11 or V_H_12 sequences, PtC liposomes or PC‐BSA, also gave a lower percentage of staining of CLL‐like cells of *Siglecg* ^−/−^ TCL1 mice. Also, in the SV40 largeT CLL model a lower percentage of V_H_11 usage was detected in *Siglecg* ^−/−^ mice, compared to transgenic controls (Pal Singh *et al*, 2018). In normal *Siglecg* ^−/−^ mice a change in the IgV_H_ repertoire has been previously observed, with less V_H_11 and V_H_12 usage and less PtC‐ and less PC‐binding BCRs on their B1a cells than in WT controls (Jellusova *et al*, 2010). All these data suggest, that earlier onset and more severe cause of the CLL‐like disease occurs in *Siglecg* ^−/−^ TCL1 mice, despite the reduced usage of typical IgV_H_ sequences of CLL‐like cells. Thus, these typical V_H_ sequences may not be crucial for the severity of the disease. It was previously shown that Siglec‐G deficiency leads to a better survival of B1a cells (Jellusova *et al*, 2010), a factor which may also contribute to the mechanism of stronger expansion of CLL‐like cells.

In human CLL patients we found a downmodulation of the human Siglec‐G orthologue Siglec‐10. Siglec‐10 was about twofold downmodulated on CLL cells compared to residual B cells of the patients. Also, data from mRNA sequencing of CLL cohorts did show a downmodulation of *SIGLEC10*. The lower mRNA expression of *SIGLEC10* in CLL cells may directly cause the lower surface expression, however posttranslational mechanism such as downmodulation of Siglec‐10 protein from the surface of CLL cells cannot be excluded. Siglecs are known to be involved in endocytosis of sialic acid carrying ligands and are cycling receptors (O'Reilly *et al*, 2011). On human CLL cells there may be a selective pressure to downmodulate this inhibitory receptor from the surface to increase BCR signaling. Similarly, it has been described that the inhibitory receptor CD22 (Siglec‐2) is expressed at lower levels on CLL cells than on normal naïve human B cells (Jasper *et al*, 2011; Salem & Stetler‐Stevenson, 2019). We are not suggesting that downmodulation of Siglec‐10 from the surface of human CLL cells is mechanistically involved in the development of CLL, but the parallel data from Siglec‐G in the mouse CLL model suggest that downmodulation of this inhibitory receptor may support the maintenance or severity of the disease.

The findings of the Siglec‐G overexpressing mice suggest that targeting of Siglec‐G/ Siglec‐10 with agents that increase the inhibitory function of this receptor may be a novel treatment option for CLL. Of note, the Siglec‐10 expression on human CLL cells is just about twofold diminished, suggesting that the expression level is still sufficient to target this surface protein. One way may be agonistic antibodies. One such example is epratuzumab, an anti‐CD22 antibody which triggers inhibition of BCR mediated signaling and was tested in clinical trials for the autoimmune disease SLE (Leonard & Goldenberg, 2007; Wallace *et al*, 2014; Özgör *et al*, 2016). Another possibility may be synthetic ligands, derived from the natural Siglec ligands sialic acids. High‐affinity synthetic ligands for CD22 have been developed in monomeric, oligomeric forms or coupled to nanoparticles (Macauley *et al*, 2013; Prescher *et al*, 2014; Bull *et al*, 2016). Such synthetic ligands could also be screened for agonistic stimulations of Siglec‐G and Siglec‐10 *in vitro* or in the CLL mouse model *in vivo*.

## Materials and Methods

### Mouse models

To study the role of Siglec‐G in CLL, Siglec‐G knockout (*Siglecg* ^−/−^) mice (BALB/c background) (Hoffmann *et al*, 2007) were crossed to Eμ‐TCL1 mice (BALB/c background; mice were kindly provided by E. Hobeika and H. Jumaa, University of Ulm) (Bichi *et al*, 2002). As a control wild type (WT) BALB/c and *Siglecg* ^−/−^ mice were chosen as littermate controls or were age matched from *Siglecg* ^−/−^ × Eμ‐TCL1 crosses. For the generation of Siglec‐G overexpressing (*Siglecg*‐R26^ki/ki^ × mb1^cre^) mice the cDNA of Siglec‐G was cloned into the target vector pROSA26 (generous gift of A. Gessner, University of Regensburg), between the short (SA) and the long arm (LA) of the ROSA26 (R26) locus. This vector also contained a CAG enhancer cassette, which consists of the chicken ß‐actin promoter and the early enhancer element of cytomegalovirus. To allow cell type‐specific protein overexpression a transcriptional stop cassette flanked by two loxP sites in front of the Siglec‐G cDNA, was used. The target vector was then introduced into BalbI embryonic stem cells (Noben‐Trauth *et al*, 1996), checked for homologous recombination and the knockin mice were generated by blastocyst injection. *Siglecg*‐R26^ki/ki^ mice were crossed with mb1‐cre mice (BALB/c background) to obtain B‐cell specific overexpression of *Siglecg*. Mice were kept in the animal facilities of Friedrich‐Alexander‐University Erlangen‐Nuremberg under specific pathogen‐free conditions in individually ventilated cages. All experiments were performed in accordance with the German law for protection of animals, after approval by the animal welfare committee.

### Cell preparation and flow cytometry

Single‐cell suspensions of bone marrow, spleen, liver, lymph nodes (cervical and inguinal) or isolated cells of peritoneal lavage were prepared in PBS (Life Technologies) and treated with 1 M ammonium‐chloride‐potassium lysis buffer to deplete erythrocytes. The liver was perfused with PBS, then predigested with collagenase D (0.05 U/ml; from Clostridium histolyticum; Roche) and DNase I (conc 0.03 mg/ml; DNase I from bovine pancreas; Roche) in HBSS for 30 min at 37°C. Cells were stained for 30 min at 4°C using the following antibodies: APC‐anti‐B220 (BioLegend; RA3‐6B2), PE‐anti‐CD5 (BD; 53–7.3), APC‐anti‐CD5 (eBioscience; 53–7.3), BrV421‐anti‐CD19 (1D3; BD), FITC‐anti‐CD45 (BioLegend; 30‐F11), BrV421‐anti‐IgM (BD; R6‐60.2), Fc‐block (2.4G2, hybridoma purified in our laboratory), Phosphatidylcholine (PtC)‐ liposomes‐FITC (FormuMax Scientific) and Phosphorylcholine (PC)‐BSA‐FITC (BioSearch Technologies). Fixable viability dye (eBioscience) was used to stain dead cells and exclude them from analysis. The staining was performed in PBS containing 0.1% BSA (Carl Roth), 2 mM EDTA (Carl Roth), and 2 mM sodium azide (Sigma‐Aldrich). After the staining cells were washed with the staining buffer and fixed with 2% PFA in PBS. All centrifugation steps were performed at 4°C and 300 *g* for 5 min. Data was acquired via Cytoflex S (Beckman Coulter) flow cytometer and analyzed using FlowJo software (TreeStar). Total cell numbers of living cells were determined by trypan blue staining.

### Blood preparation

Blood was obtained by incision of the tail vein or by puncturing of the heart. For FACS analysis blood was collected in PBS containing 1% heparin (Ratiopharm), followed by erythrocyte depletion as described above. For hematological analysis, blood was collected in EDTA coated tubes (MiniCollect; Greiner) and analyzed by the hematology system ADVIA 120 (Siemens).

### Calcium mobilization assay

About 2 × 10^6^ to 2 × 10^7^ cells from blood, peritoneal cavity and spleen were resuspended in 0.7 ml RPMI 1640 media (Life Technologies) containing 5% FCS (PAN Biotech), loaded with 0.7 mM indo‐1‐AM pluronic acid F‐127 (Molecular Probes) and incubated for 25 min at 30°C upon shaking. Subsequently, 0.7 ml RPMI 1640 media (Life Technologies) containing 10% FCS (PAN Biotech) were added and the cells were incubated for another 10 min at 37°C. Cells were washed twice and stained extracellularly with FITC‐anti‐B220 (BioLegend; RA3‐6B2) and APC‐anti‐CD5 (eBioscience; 53–7.3) as described above. The cells were then washed and resuspended in Krebs‐Ringer solution to measure calcium mobilization using LSR II (Becton Dickinson). First the basal Ca^2+^ level was determined for 50 s. Then the BCR was stimulated by 3.25, 6.5 or 13 μg anti‐IgM [F(ab)_2_] (Jackson ImmunoResearch) and the sample was measured up to 3 min. The Indo‐1 loading efficiency was determined by separate ionomycin (Sigma‐Aldrich) stimulation. The data was analyzed with FlowJo software (Tree Star).

### Antibody repertoire sequencing (Ig‐seq) sample preparation

In order to obtain information on the BCR repertoire of different mice, blood was drawn and prepared as described above at defined time points. Time points were chosen at the age of 12, 24, 36, and 48 weeks for TCL1, TCL1 × *Siglecg* ^−/−^ mice and their corresponding controls and at the age of 28, 36, and 48 weeks for TCL1 × *Siglecg*‐R26^ki/ki^ and TCL1 × *Siglecg*‐R26^ki/ki^ × mb1^cre^ mice and controls. Cells were stained with FITC‐anti‐CD19 (BioLegend, 1D3) for 30 min at 4°C. After incubation cells were washed with the nucleic acid binding dye 4′6‐diamino‐2‐phenylindol (DAPI) diluted 1:250 in PBS containing 0.5% FCS and 2 mM EDTA. CD19‐positive and DAPI‐negative cells were separated using a FACS Aria III cell sorter (Becton Dickinson) and collected in RLT buffer (RNeasy Plus Micro Kit; Qiagen). Subsequently, RNA isolation was performed using the RNeasy Plus Micro Kit from Qiagen according to the manufacturer's protocol and samples were stored at −80°C.

### Ig‐seq library preparation by molecular amplification fingerprinting

The method molecular amplification fingerprinting (MAF) was done similarly as published before (Werner *et al*, 2022). The method consists of assigning unique molecular identifiers (UIDs) to individual transcripts before and during multiplex PCR to minimize possible errors arising in library preparation (Khan *et al*, 2016). The Illumina MiSeq platform was used for sequencing with a pair of overlapping paired‐end reads (2 × 300 bp) and 20% PhiX. Quality was assured by FastQC analysis, PhiX‐QC and UMI error correction (Werner *et al*, 2022). Paired‐end sequences were merged with the pRESTO toolkit. After annotation via IMGT/HighV‐QUEST the NGS data was analyzed with the ARGALAXY pipeline.

### Flow cytometric analysis of phosphoproteins

Spleen cells were prepared and stained extracellularly for APC‐anti‐B220 (BioLegend; RA3‐6B2) and PE‐anti‐CD5 (BD; 53–7.3) as described above. Intracellular staining was performed using the ADG Fix&Perm Kit (Dianova). After the initial staining cells were washed with PBS, then fixed with paraformaldehyde‐containing buffer A for 10 min at room temperature followed by washing with PBS. Antibodies for intracellular staining were dissolved in saponin‐containing buffer B and the staining was performed for 30 min at room temperature. Subsequently, cells were first washed with PBS, then with PBS containing 0.1% BSA (Carl Roth), 2 mM EDTA (Carl Roth) and 2 mM sodium azide (Sigma‐Aldrich). The following antibodies were applied for intracellular staining: AF488‐anti‐Akt (pS473) (BD; M89‐61), AF488‐anti‐Btk (pY551)/Ikt (pY511) (BD; 24a), AF488‐anti‐PLC‐γ2 (pY759) (BD; K86‐689.37), AF488‐anti‐S6 (pS240) (BD; N4‐41), AF488‐anti‐S6 (pS235/236) (BD; N7‐548), AF488‐anti‐ZAP70 (pY319)/Syk (Y352) (BD; 17A/P‐ZAP70), purified hamster anti‐mouse BCL‐2 (BD; 3F11) with secondary antibody anti‐Armenian Hamster‐AF488 (Jackson Immunoresearch). AF488‐anti‐Mouse IgG1κ isotype control (BD; MOPC‐21) and purified armenian hamster IgG1κ isotype control (BD) were used.

### Human samples

PB samples were obtained after written informed consent was received from participants prior to inclusion in the study according to the Declaration of Helsinki, and approval by the ethics committee of the Medical Faculty at the University of Duisburg‐Essen, Germany (BO‐10‐4380). B cells were isolated by Ficoll density centrifugation (density 1.077 g/ml, Pan BioTech, Aidenbach, Germany) followed by staining with anti‐CD3 (BD Biosciences, Heidelberg, Germany), anti‐CD5 (BioLegend, Koblenz, Germany), anti‐CD20, anti‐CD23, and anti‐CD27 (each BD Biosciences) antibodies. Stained cells were analyzed on a CytoFLEX S flow cytometer (Beckman Coulter, Krefeld, Germany) using CytExpert v2.4 (Beckman Coulter) or FlowJo v10.6.2 (BD Biosciences) software. RNAseq data were retrieved from (Budeus *et al*, 2021).

### Statistical analysis

If not stated otherwise in the figure legend data are presented as mean ± SD. Data were analyzed and illustrated using GraphPad Prism software (GraphPad Software Inc., San Diego, CA). Normal distribution was tested with Shapiro Wilk normality test. Normally distributed data were then analyzed by ordinary one‐way ANOVA followed by Šídák's multiple comparison test. Non‐parametric distribution was tested using Kruskal–Wallis test with Dunn's *post hoc* test. Differences in survival were analyzed with Kaplan–Meier estimates and the groups were compared for significance via the log‐rank test. *P* < 0.05 was considered significant.

## Author contributions

**Bettina Röder:** Conceptualization; investigation; writing – original draft. **Hannah Fahnenstiel:** Conceptualization; investigation. **Simon Schäfer:** Formal analysis. **Bettina Budeus:** Investigation. **Maria Dampmann:** Investigation. **Melanie Eichhorn:** Investigation. **Sieglinde Angermüller:** Investigation. **Claudia Brost:** Investigation. **Thomas H Winkler:** Formal analysis; supervision. **Marc Seifert:** Formal analysis; supervision. **Lars Nitschke:** Conceptualization; funding acquisition; writing – original draft; writing – review and editing.

## Disclosure and competing interests statement

The authors declare that they have no conflict of interest.

## Supporting information



Appendix

Expanded View Figures PDF

Source Data for Figure 1

PDF+

Source Data for Figure 2

Source Data for Figure 4

Source Data for Figure 5

Source Data for Figure 7

Source Data for Figure 8

## Data Availability

The mRNA sequencing data of Figs 3 and 6 are available at: gene expression omnibus (http://www.ncbi.nlm.nih.gov/geo/query/acc.cgi?acc=GSE227678) under GEO ID: GSE227678.
