# Supplementary material for: The inhibitory receptor Siglec‐G controls the severity of chronic lymphocytic leukemia
Source: EMBO Rep. 2023 Jul 10;24(8):e56420. doi: 10.15252/embr.202256420 (PMC10398647; doi:10.15252/embr.202256420)
Supplement: Supplementary file 1 — Appendix [file EMBR-24-e56420-s009.pdf]

Table of content

Appendix Fig. S1	page 1
Appendix Fig. S2	page 2
Appendix Fig. S3	page 3
Appendix Fig. S4	page 4
Appendix Fig. S5	page 5
Appendix Table S1	page 6

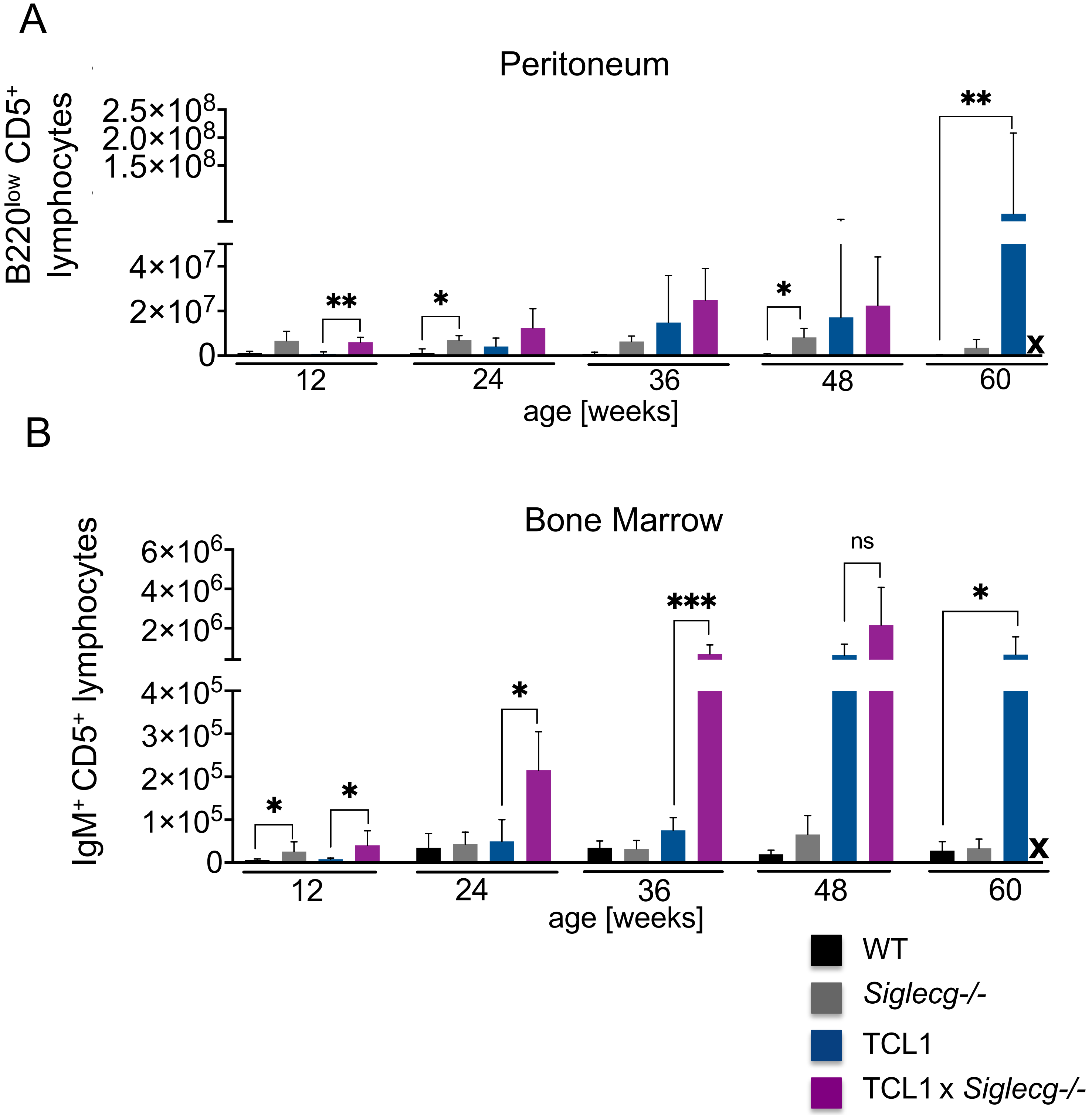

Appendix Figure S1. Earlier infiltration of CLL-like cells in peritoneal cavity and bone marrow in TCL1 x *Sigelcg-/-* mice.

Depicted are the absolute cell numbers of gated B220^{low} CD5^{+} lymphocytes in the peritoneal cavity (A) or IgM^{+} CD5^{+} cells the bone marrow (B), as mean values with ±SD. Cell were pre-gated on single and living cells. Significant differences between groups were tested either by ordinary one-way ANOVA with Šídák's post-hoc test if there was a normal distribution, or by one-way ANOVA with Kruskal-Wallis test and corrected for multiple comparison with Dunn's test if there was no overall normal distribution. The different time points were tested seperatly for significance. *p<0.05, **p<0.01, ***p<0.001. n=5-12 animals per genotype and time point, summarized from ar least 10 indepemdent experiments.

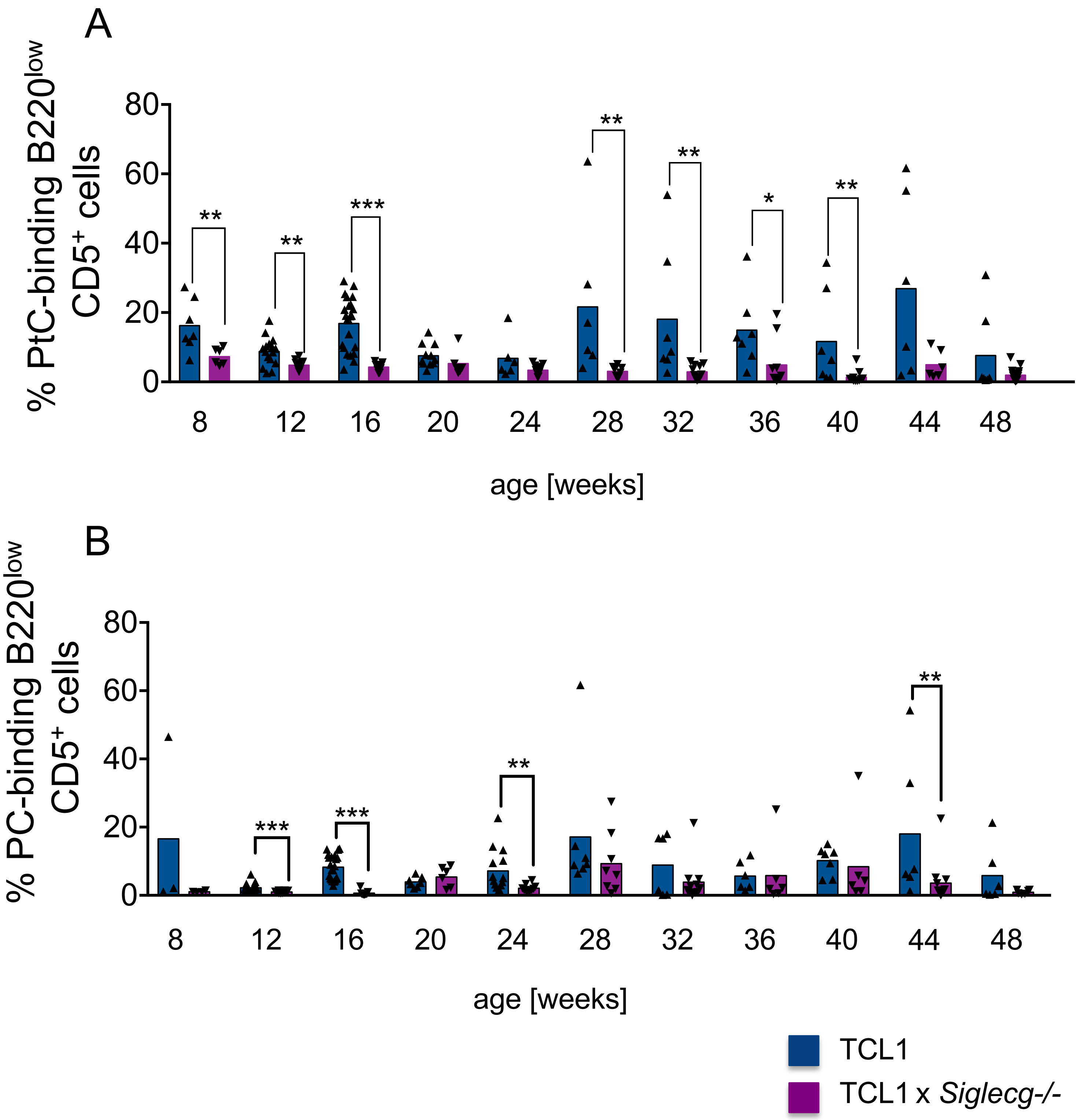

Appendix Figure S2. Less binding of PC- or PtC-containing antigens in CLL-like cells of TCL1 x *Siglecg-/-* mice.
Blood was collected every 4 weeks and analysed by flow cytometry. The diagrams show the percentage of (B) PtC-liposomes or (C) PC-BSA binding cells of the selected $B220^{low}CD5^{+}$ population over a period of 48 weeks. The mean value is shown and each dot represents one mouse. The significance of the differences was determined using the Mann-Whitney test, *p<0.05, **p<0.01, ***p<0.001. n= 6-23 animals per time point and genotype, summarized from at least 20 independent experiments.

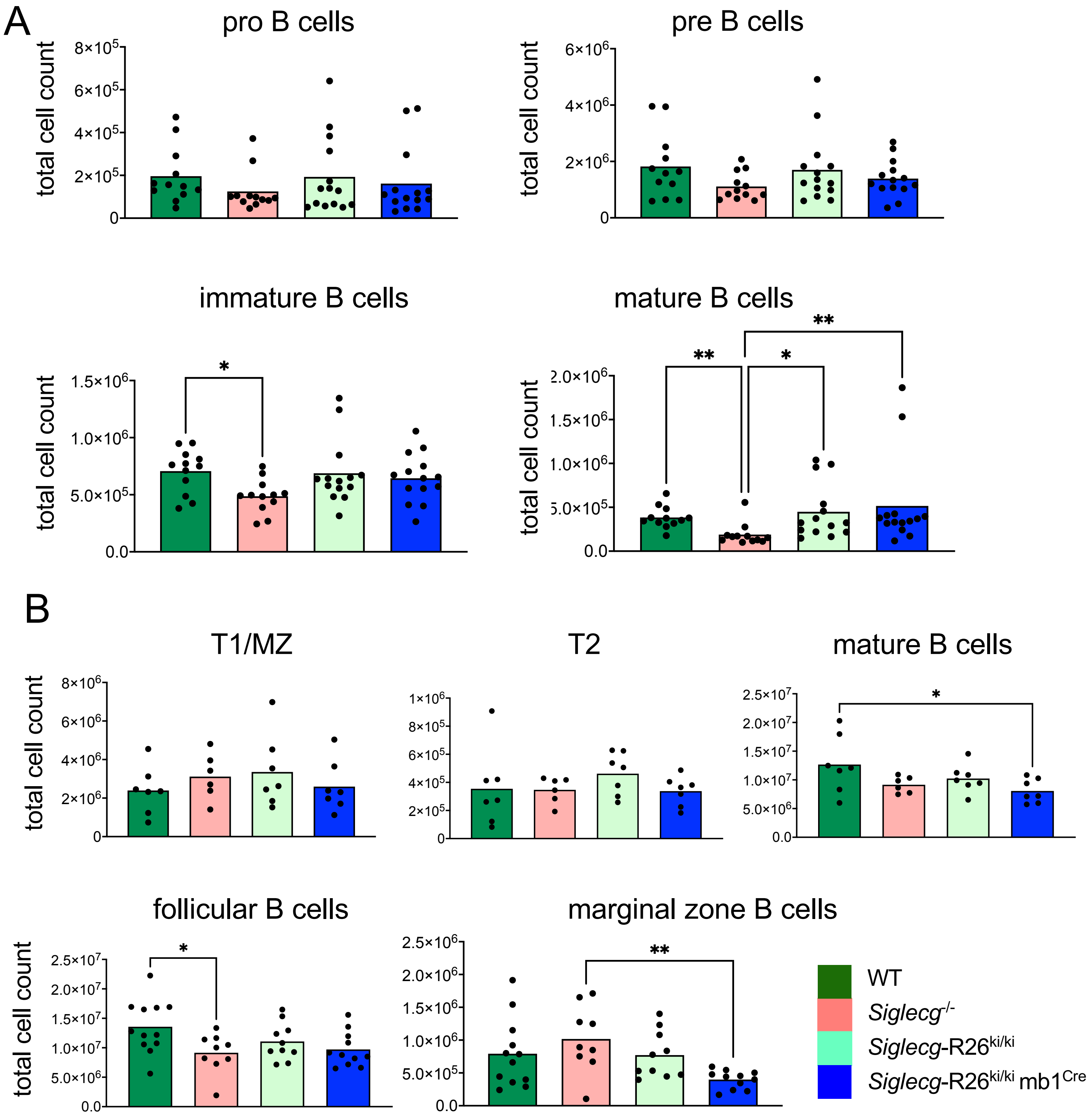

Appendix Figure S3: Siglec-G overexpression has no impact on B cell development and maturation. Depicted are the absolute cell numbers of different cell populations analysed by flow cytometry A) in the bone marrow and B) spleen. Cell were pre-gated on single and living lymphocytes.
Gating of B cell populations in A) as follows: pro B cells ($B220^{+}$, c-kit^{+} $CD25^{-}$), pre B cells ($B220^{+}$, $CD25^{+}$), immature B cells ($B220^{med}$, IgM^{low}), mature B cells ($B220^{+}$, IgM^{low})
and in B): T1/MZ ($B220^{+}$, IgM^{hi}) T2 ($B220^{+}$, IgM^{hi}, IgD^{hi}), mature B cells ($B220^{+}$, IgD^{+}, IgM^{med}), follicular ($B220^{+}$, $CD23^{med}$, $CD21^{low}$) and marginal zone B cells ($B220^{+}$, $CD23^{low}$, $CD21^{hi}$).
Significant differences between groups were tested either by ordinary one-way ANOVA with Šídák's post-hoc test if there was a normal distribution, or by one-way ANOVA with Kruskal-Wallis test and corrected for multiple comparison with Dunn's test if there was no overall normal distribution.. *$p<0.05$, **$p<0.01$, ***$p<0.001$. n=6-14 animals per genotype

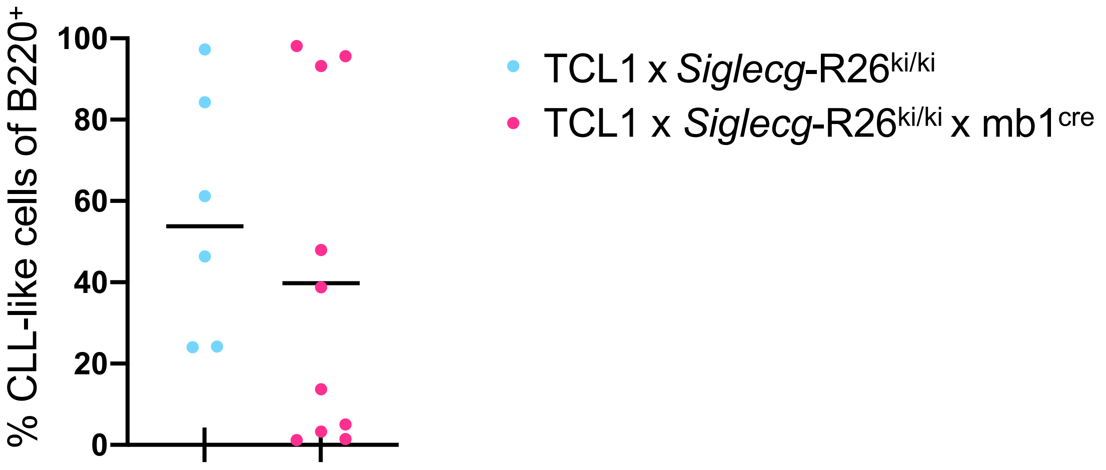

Appendix Figure S4. Percentages of CLL-like cells in TCL1 x *Siglecg*-R26$^{ki/ki}$ and TCL1 x *Siglecg*-R26$^{ki/ki}$ x mb1^{cre} mice.
Scatter dot plot shows percentages of CLL-like cells for the intracellular phosphoprotein analysis in Fig.8B.

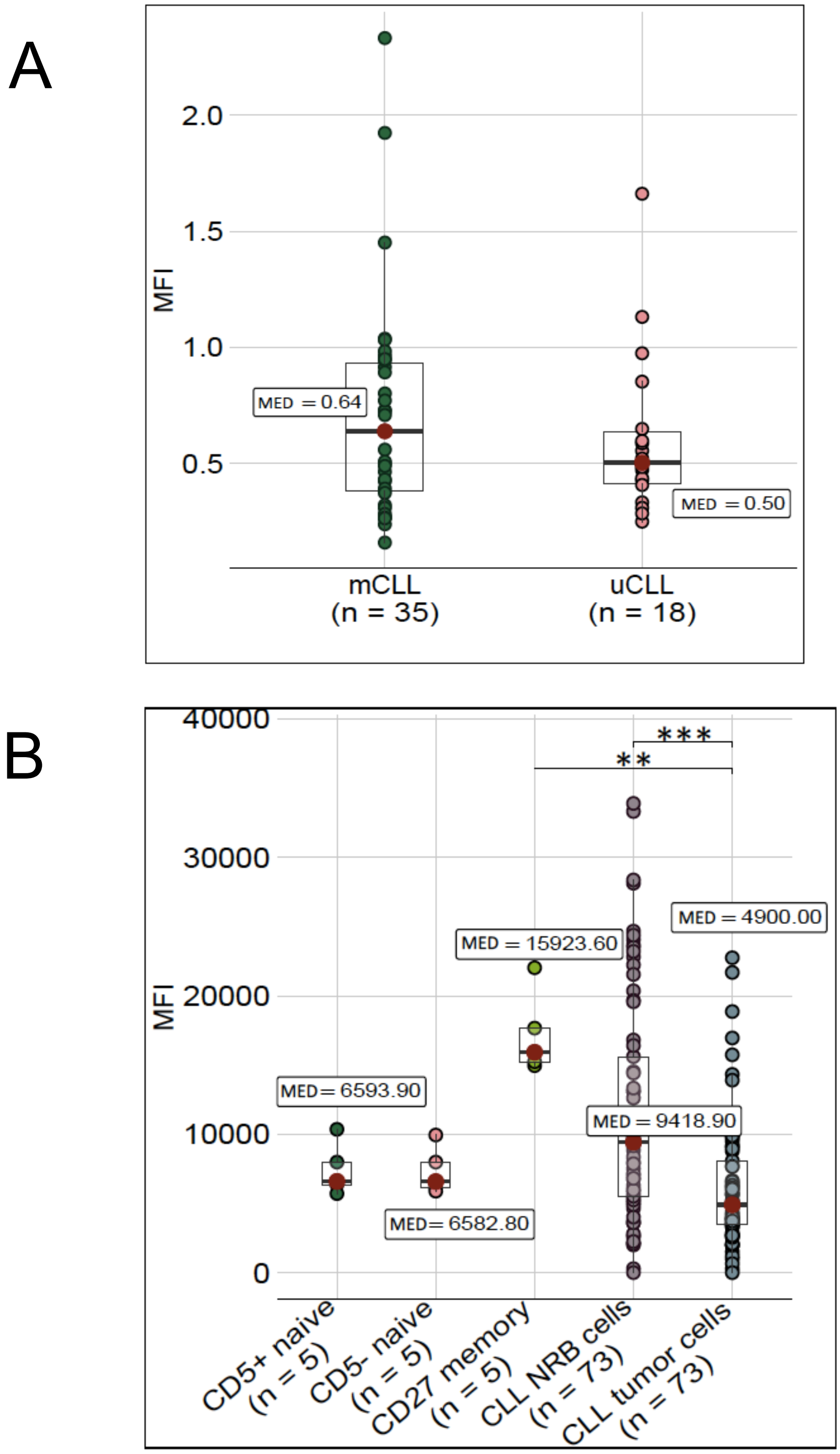

Appendix Figure S5. Relative downmodulation of surface Siglec-10 on CLL cells and expression of Siglec-10 on CLL cells compared to normal human B cells .
A) The fold-change downmodulation of Siglec-10 is given per each sample by providing the quotient of median fluorescence intensities (MFI) from the CLL tumor cell population divided by the normal residual B cell population shown in Fig.9A. Data information: Wilcoxon rank sum test, not significant. Samples are biological replicates
B) The mean fluorescence intensity (MFI) of surface Siglec-10 is given from 73 CLL cases (including 20 cases with unknown IgV-mutation status), always including tumor cells ($CD20^{low}CD5^{high}$) and normal residual B (NRB) cells ($CD20^{high}CD5^{-}$) as paired samples, and from five healthy age-matched donors, discriminating naïve ($IgD^{high}CD27^{-}CD5^{-}$), mature $CD5^{+}$ ($IgD^{+}CD27^{-}CD38^{low}$) and $CD27^{+}$ memory B cell subsets ($IgM/IgG/IgA^{+}CD27^{+}$). Wilcoxon rank sum test, **p<0.01, ***p<0.001. Median as central band, box encompassed from first to third quartile, whiskers are the smallest or largest value no further than 1.5 * IQR (range from first to third quartiles) from the hinge.

A

ID	genotype	V_H	D	J_H	HCDR3 length	N nucleotide additions	P nucleotide additions	mutation rate
T7551	TCL1	3-2	D2-4	J2	12	10	1	0.5 %
T6006	TCL1 x *Siglecg-/-*	5-6	D3-1	J3	15	6	4	2.4 %
T7616	TCL1 x *Siglecg-/-*	3-2	D1-1	J3	14	3	0	1 %
T7620	TCL1 x *Siglecg-/-*	2-6-5	D2-14	J3	6	6	1	0 %

B

ID	genotype	V_H	D	J_H	HCDR3 length	N nucleotide additions	P nucleotide additions	mutation rate
U1515	TCL1 x *Siglecg*-R26 $^{ki/ki}$	1-67	D1-1	J2	11	8	3	3.7 %
U1662	TCL1 x *Siglecg*-R26 $^{ki/ki}$	12-3	D1-1	J1	12	0	0	3.7 %
U2517	TCL1 x *Siglecg*-R26 $^{ki/ki}$	11-2	D2-1	J1	11	0	0	3.1 %

Appendix Table S1. Details about the clonal IgV_H sequences from CLL-like cells shown in Fig.3 and Fig.6.
V_H, D and J_H usages, CDR3 length, N and P nucleotide addition and V_H mutation rate of the monoclonal Ig sequences of CLL-like cells from Fig.3 are shown in A) and from Fig.6 are shown in B).
