# Supplementary material for: The inhibitory receptor Siglec‐G controls the severity of chronic lymphocytic leukemia
Source: EMBO Rep. 2023 Jul 10;24(8):e56420. doi: 10.15252/embr.202256420 (PMC10398647; doi:10.15252/embr.202256420)
Supplement: Supplementary file 2 — Expanded View Figures PDF [file EMBR-24-e56420-s005.pdf]

Expanded View Figures

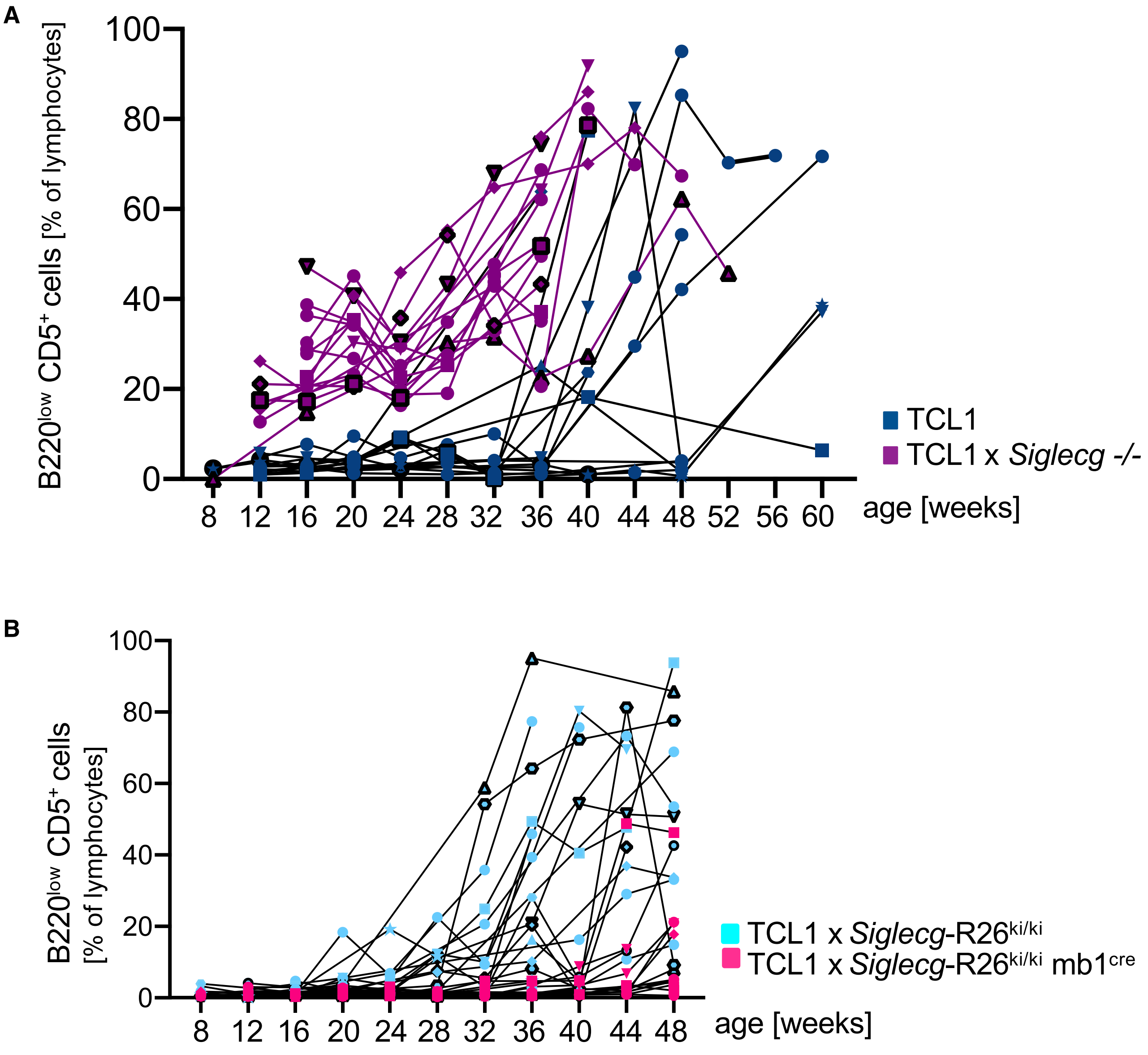

Figure EV1. Earlier expansion of the CLL-like cell population in TCL1 × Siglecg$^{-/-}$ mice and later expansion in TCL1 × Siglecg-R26$^{ki/ki}$ mb1^{cre} mice, shown for individual mice over time.

A, B Shown are percentages of B220^{low} CD5^{+} cells in the blood of individual mice over time in (A) comparison of TCL1 and TCL1 × Siglecg$^{-/-}$ mice and in (B) TCL1 × *Siglecg*-R26$^{ki/ki}$ and TCL1 × *Siglecg*-R26$^{ki/ki}$ mb1^{cre} control mice. To distinguish different mice different symbols were used to represent individual mice that are connected by lines. *N* = 15 TCL1; *n* = 15 TCL1 × *Siglecg*$^{-/-}$; *n* = 22 TCL1 × *Siglecg*-R26$^{ki/ki}$; *n* = 17 TCL1 × *Siglecg*-R26$^{ki/ki}$ mb1^{cre}.

 © 2023 The Authors

Figure EV2. Earlier signs of leukocytosis and lymphocytosis in TCL1 × *Siglecg*$^{-/-}$ mice.

A–D For hematological analysis of blood cells (A) the leukocyte count (B) the lymphocyte count (C) the platelet count and (D) the red blood cell count were determined with an Advia 120 hematology analysis machine. The mean values are shown with SD. Significant differences between groups were determined by one-way ANOVA with Kruskal–Wallis and corrected for multiple comparison with Dunn's test, *$P < 0.05$, **$P < 0.01$, ***$P < 0.001$. $n = 5$–16 animals per time point and genotype, summarized from at least five independent experiments.

© 2023 The Authors

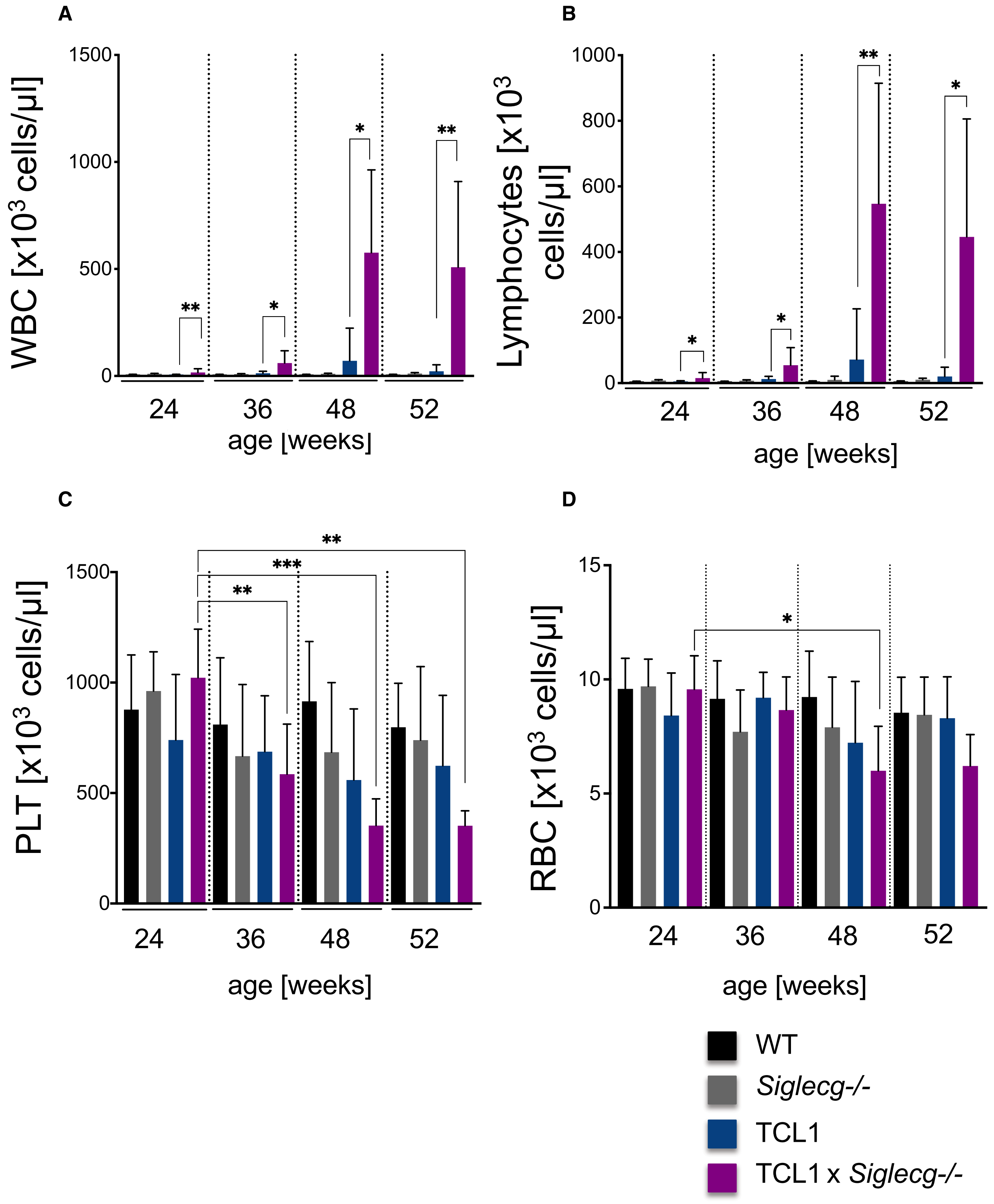

Figure EV2.

 © 2023 The Authors

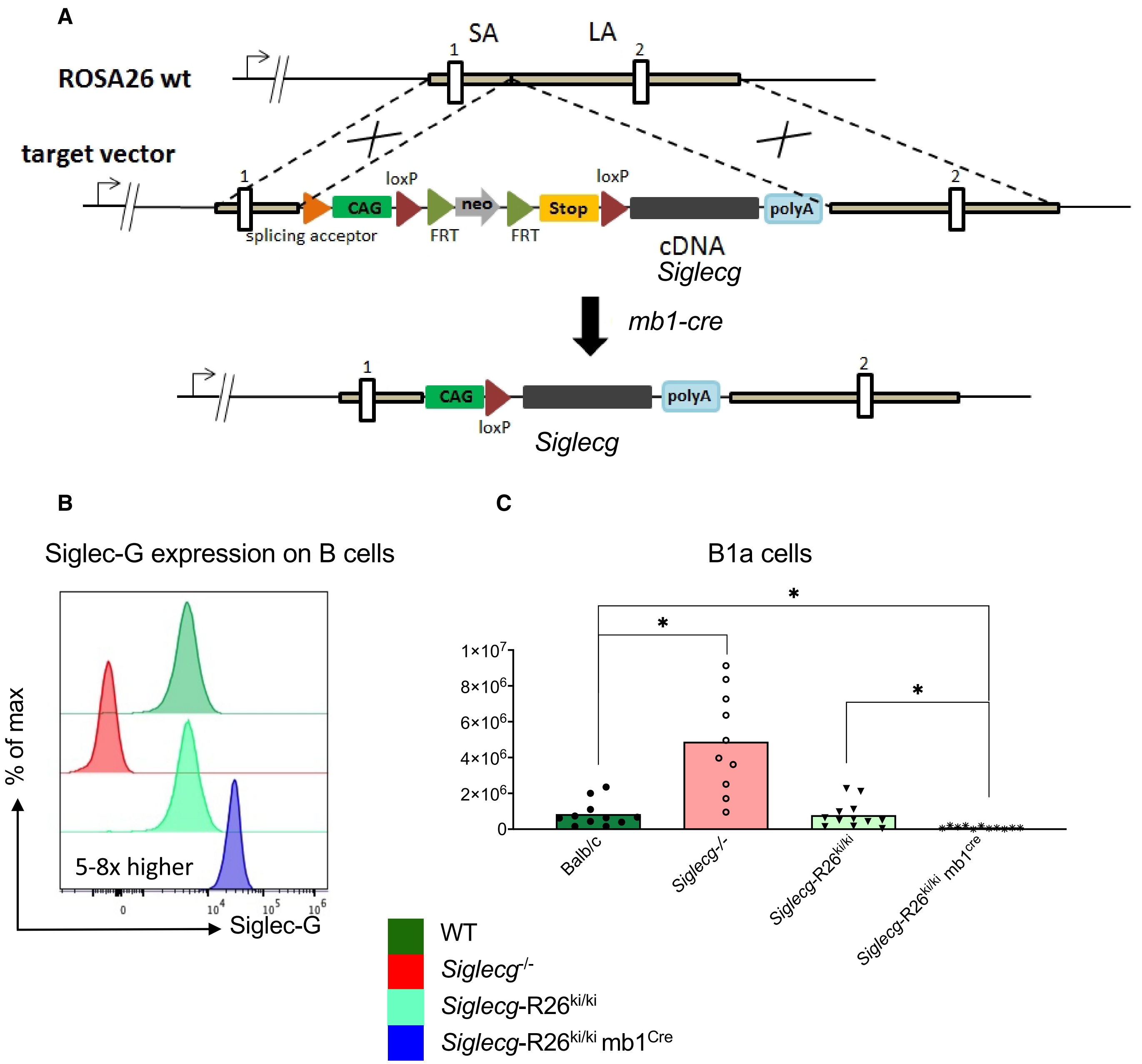

Figure EV3. Generation of Siglec-G overexpressing mice.

A For the generation of Siglec-G overexpressing mice the cDNA of Siglec-G was cloned into the target vector, between the short (SA) and the long arm (LA) of the ROSA26 (R26) locus. Overexpression was facilitated by the CAG enhancer cassette, which contains the chicken ß-actin promoter and the early enhancer element of cytomegalovirus. A transcriptional stop cassette flanked by two loxP sites ensured B cell-specific expression by mating with the mb1cre mouse strain. The cre recombinase specifically removes the stop cassette so that the enhancer cassette is brought in front of the cDNA and transcription of the Siglec-G cDNA occurs.

B Siglecg-R26$^{ki/ki}$ mb1^{cre} mice showed a five to eightfold overexpression of Siglec-G on the surface of mature B cells in the spleen and peritoneal cavity, respectively. The histograms represent the expression of Siglec-G on conventional B2 cells of the spleen.

C In the peritoneal cavity a reduction of B1a cells was observed in Siglecg-R26$^{ki/ki}$ mb1^{cre} mice in contrast to the enlargement of this population in Siglecg$^{-/-}$ mice. Shown are the mean values of the absolute cell counts. Cells were pre-gated for single, living lymphocytes subsequently, B1a cells were identified as B220^{low} and CD5^{+}. Significant differences between groups were determined with Kruskal–Wallis and corrected for multiple comparison with Dunn's test, $^{*}P < 0.05$. $n = 6$–14 animals per genotype; every dot represents a mouse. Data are summarized from 5 independent experiments.

© 2023 The Authors

Figure EV4. No leukocytosis or lymphocytosis in TCL1 × *Siglecg*-R26$^{ki/ki}$ × mb1^{cre} mice.

A–D For hematological analysis of blood cells (A) the leukocyte count (B) the lymphocyte count (C) the platelet count and (D) the red blood cell count were determined with an Advia 120 hematology analysis machine. The mean values are shown with ± SD. Significant differences between groups were determined by one-way ANOVA with Kruskal–Wallis and corrected for multiple comparison with Dunn's test, $^{*}P < 0.05$, $^{**}P < 0.01$. $n = 5$ animals per time point and genotype, summarized from at least 5 independent experiments.

 © 2023 The Authors

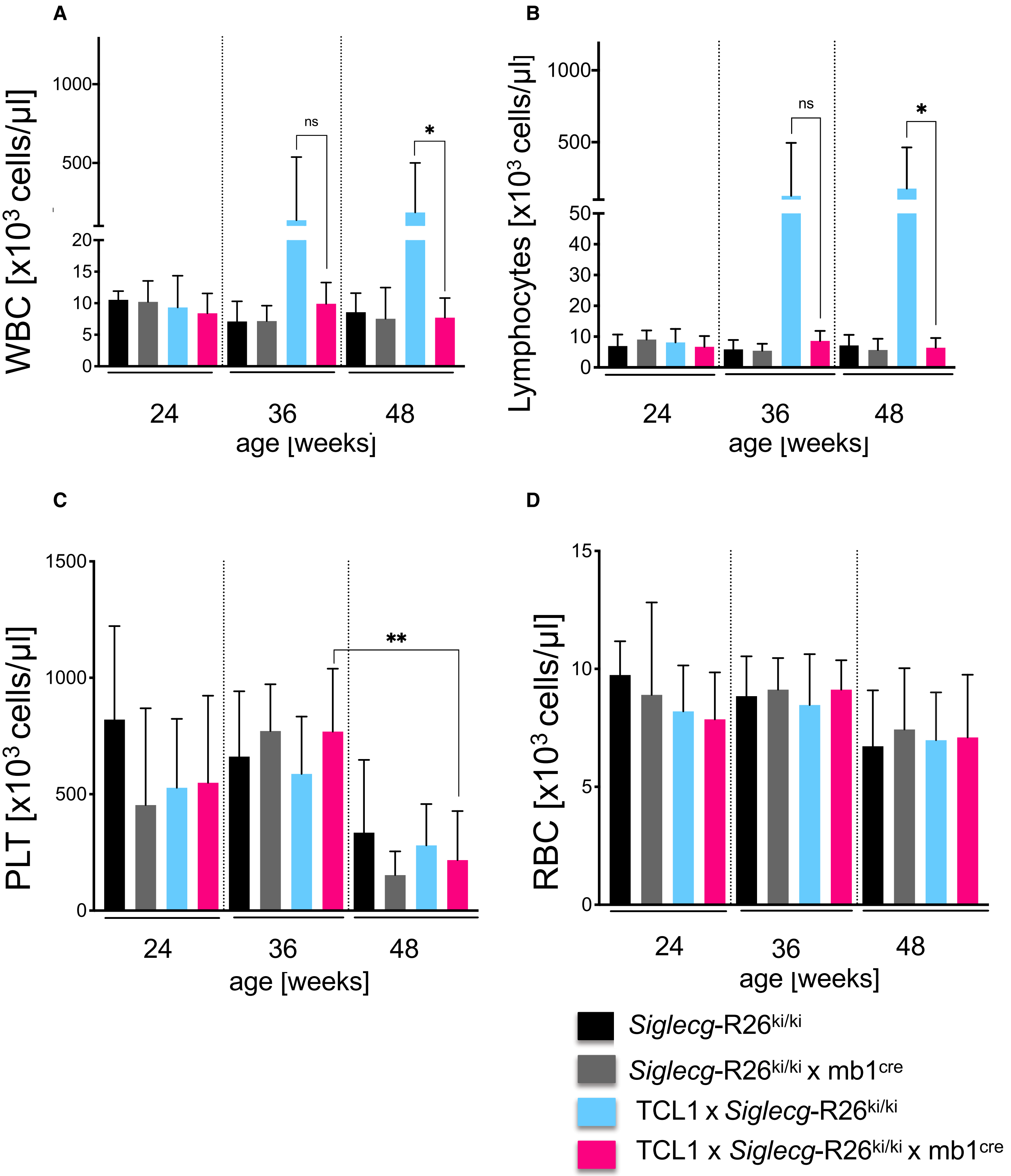
A
WBC [x10³ cells/µl]
1000
500
20
15
10
5
0
ns
*
24
36
48
age [weeks]
B
Lymphocytes [x10³ cells/µl]
1000
500
50
40
30
20
10
0
ns
*
24
36
48
age [weeks]
C
PLT [x10³ cells/µl]
1500
1000
500
0
**
24
36
48
age [weeks]
D
RBC [x10³ cells/µl]
15
10
5
0
24
36
48
age [weeks]
Siglecg-R26ki/ki
Siglecg-R26ki/ki x mb1cre
TCL1 x Siglecg-R26ki/ki
TCL1 x Siglecg-R26ki/ki x mb1cre

Figure EV4.

Figure EV5. Downmodulation of human Siglec-10 surface expression on CLL cells compared to normal residual B cells.

A Peripheral blood B cells, pre-gated on single, living B lymphocytes ($CD19^{+}$). One representative plot for the gating of CLL cells ($CD20^{low}CD5^{high}$) and normal residual (NRB) cells ($CD20^{high}CD5^{-}$) is given.

B Data as in Fig 8A, separated by IgV-mutation status (mCLL and uCLL, top and bottom row, respectively) and Binet A versus Binet B or C stage of the patient (left versus right column).

Data information: Wilcoxon signed-rank test, *$P < 0.05$, ***$P < 0.001$. Samples are paired biological replicates. Median as central band, box encompassed from first to third quartile, whiskers are the smallest or largest value no further than 1.5 * IQR (range from first to third quartiles) from the hinge.

 © 2023 The Authors

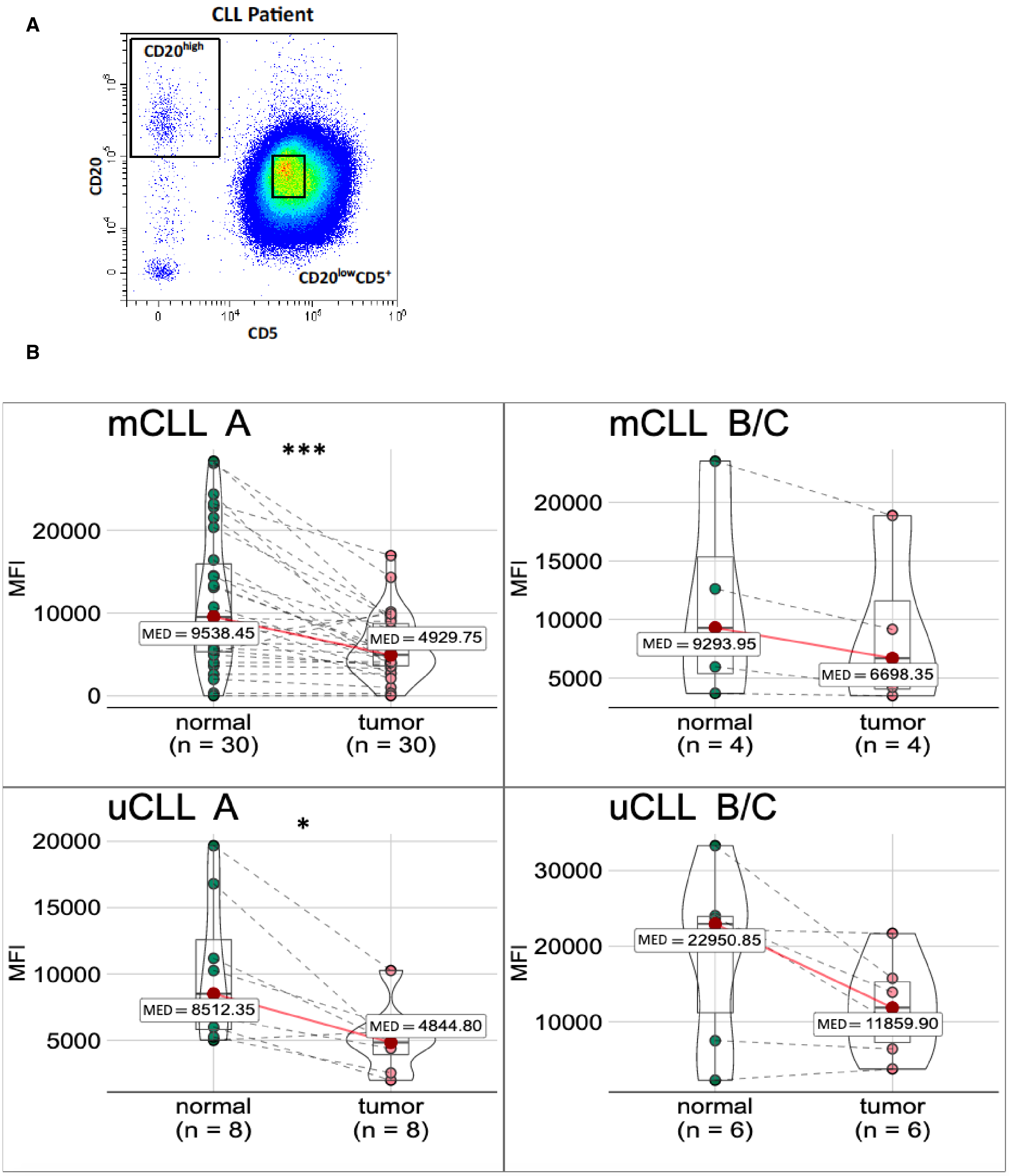
A
CLL Patient
CD20high
CD20lowCD5+
CD20
CD5
B
mCLL A
***
MFI
20000
10000
0
MED = 9538.45
MED = 4929.75
normal
(n = 30)
tumor
(n = 30)
mCLL B/C
MFI
20000
15000
10000
5000
MED = 9293.95
MED = 6698.35
normal
(n = 4)
tumor
(n = 4)
uCLL A
*
MFI
20000
15000
10000
5000
MED = 8512.35
MED = 4844.80
normal
(n = 8)
tumor
(n = 8)
uCLL B/C
MFI
30000
20000
10000
MED = 22950.85
MED = 11859.90
normal
(n = 6)
tumor
(n = 6)

Figure EV5.
