# Supplementary material for: The inhibitory receptor Siglec‐G controls the severity of chronic lymphocytic leukemia
Source: EMBO Rep. 2023 Jul 10;24(8):e56420. doi: 10.15252/embr.202256420 (PMC10398647; doi:10.15252/embr.202256420)
Supplement: Supplementary file 10 [file EMBR-24-e56420-s001.pdf]

EMBO reports

The inhibitory receptor Siglec-G controls the severity of chronic lymphocytic leukemia

Bettina Röder, Hannah Fahnenstiel, Simon Schäfer, Bettina Budeus, Maria Dampmann, Melanie Eichhorn, Sieglinde Angermüller, Claudia Brost, Thomas Winkler, Marc Seifert, and Lars Nitschke
DOI: 10.15252/embr.202256420

Corresponding author(s): Lars Nitschke (lars.nitschke@fau.de)

Review Timeline:

Submission Date:	3rd Nov 22
Editorial Decision:	14th Dec 22
Revision Received:	21st Mar 23
Editorial Decision:	27th Apr 23
Revision Received:	13th Jun 23
Accepted:	22nd Jun 23

Editor: Achim Breiling

Transaction Report:

(Note: With the exception of the correction of typographical or spelling errors that could be a source of ambiguity, letters and reports are not edited. Depending on transfer agreements, referee reports obtained elsewhere may or may not be included in this compilation. Referee reports are anonymous unless the Referee chooses to sign their reports.)

1st Editorial Decision 14th Dec 2022

Dear Prof. Nitschke,

Thank you for the submission of your research manuscript to EMBO reports. I have now received the reports from the three referees that were asked to evaluate your study, which can be found at the end of this email.

As you will see, the referees think that these findings are of high interest. However, they have several comments, concerns, and suggestions, indicating that a major revision of the manuscript is necessary to allow publication of the study in EMBO reports. As the reports are below, and all the referee concerns need to be addressed, I will not detail them here.

Given the constructive referee comments, I would like to invite you to revise your manuscript with the understanding that all referee concerns must be addressed in the revised manuscript and in a detailed point-by-point response. Acceptance of your manuscript will depend on a positive outcome of a second round of review. It is EMBO reports policy to allow a single round of revision only and acceptance of the manuscript will therefore depend on the completeness of your responses included in the next, final version of the manuscript.

Revised manuscripts should be submitted within three months of a request for revision. Please contact me to discuss the revision (also by video chat) if you have questions or comments regarding the revision, or should you need additional time.

When submitting your revised manuscript, please also carefully review the instructions that follow below.

PLEASE NOTE THAT upon resubmission revised manuscripts are subjected to an initial quality control prior to exposition to re-review. Upon failure in the initial quality control, the manuscripts are sent back to the authors, which may lead to delays. Frequent reasons for such a failure are the lack of the data availability section (please see below) and the presence of statistics based on n=2 (the authors are then asked to present scatter plots or provide more data points).

When submitting your revised manuscript, we will require:

1) a .docx formatted version of the final manuscript text (including legends for main figures, EV figures and tables), but without the figures included. Figure legends should be compiled at the end of the manuscript text.

2) individual production quality figure files as .eps, .tif, .jpg (one file per figure), of main figures (up to 8) and EV figures. Please upload these as separate, individual files upon re-submission.

The Expanded View format, which will be displayed in the main HTML of the paper in a collapsible format, has replaced the Supplementary information. You can submit up to 5 images as Expanded View. Please follow the nomenclature Figure EV1, Figure EV2 etc. The figure legend for these should be included in the main manuscript document file in a section called Expanded View Figure Legends after the main Figure Legends section. Additional Supplementary material should be supplied as a single pdf file labeled Appendix. The Appendix should have page numbers and needs to include a table of content on the first page (with page numbers) and legends for all content. Please follow the nomenclature Appendix Figure Sx, Appendix Table Sx etc. throughout the text, and also label the figures and tables according to this nomenclature.

For more details, please refer to our guide to authors:
http://www.embopress.org/page/journal/14693178/authorguide#manuscriptpreparation

Please consult our guide for figure preparation:
http://wol-prod-cdn.literatumonline.com/pb-assets/embo-site/EMBOPress_Figure_Guidelines_061115-1561436025777.pdf

See also the guidelines for figure legend preparation:
https://www.embopress.org/page/journal/14693178/authorguide#figureformat

3) a complete author checklist, which you can download from our author guidelines (https://www.embopress.org/page/journal/14693178/authorguide). Please insert page numbers in the checklist to indicate where the requested information can be found in the manuscript. The completed author checklist will also be part of the RPF.

Please also follow our guidelines for the use of living organisms, and the respective reporting guidelines:
http://www.embopress.org/page/journal/14693178/authorguide#livingorganisms

4) that primary datasets produced in this study (e.g. RNA-seq, ChIP-seq, structural and array data) are deposited in an appropriate public database. If no primary datasets have been deposited, please also state this in a dedicated section (e.g. 'No primary datasets have been generated and deposited'), see below.

See also: http://embor.embopress.org/authorguide#datadeposition

Please remember to provide a reviewer password if the datasets are not yet public.

The accession numbers and database should be listed in a formal "Data Availability" section (placed after Materials & Methods) that follows the model below. This is now mandatory (like the COI statement). Please note that the Data Availability Section is restricted to new primary data that are part of this study. This section is mandatory. As indicated above, if no primary datasets have been deposited, please state this in this section

Data availability

The datasets produced in this study are available in the following databases:

- RNA-Seq data: Gene Expression Omnibus GSE46843 (https://www.ncbi.nlm.nih.gov/geo/query/acc.cgi?acc=GSE46843)
- [data type]: [name of the resource] [accession number/identifier/doi] ([URL or identifiers.org/DATABASE:ACCESSION])

*** Note - All links should resolve to a page where the data can be accessed. ***

Moreover, I have these editorial requests:

6) We now request the publication of original source data with the aim of making primary data more accessible and transparent to the reader. Our source data coordinator will contact you to discuss which figure panels we would need source data for and will also provide you with helpful tips on how to upload and organize the files.

7) Our journal encourages inclusion of *data citations in the reference list* to directly cite datasets that were re-used and obtained from public databases. Data citations in the article text are distinct from normal bibliographical citations and should directly link to the database records from which the data can be accessed. In the main text, data citations are formatted as follows: "Data ref: Smith et al, 2001" or "Data ref: NCBI Sequence Read Archive PRJNA342805, 2017". In the Reference list, data citations must be labeled with "[DATASET]". A data reference must provide the database name, accession number/identifiers and a resolvable link to the landing page from which the data can be accessed at the end of the reference. Further instructions are available at: http://www.embopress.org/page/journal/14693178/authorguide#referencesformat

8) Regarding data quantification and statistics, please make sure that the number "n" for how many independent experiments were performed, their nature (biological versus technical replicates), the bars and error bars (e.g. SEM, SD) and the test used to calculate p-values is indicated in the respective figure legends (also for potential EV figures and all those in the final Appendix). Please also check that all the p-values are explained in the legend, and that these fit to those shown in the figure. Please provide statistical testing where applicable. Please avoid the phrase 'independent experiment', but clearly state if these were biological or technical replicates. Please also indicate (e.g. with n.s.) if testing was performed, but the differences are not significant. In case n=2, please show the data as separate datapoints without error bars and statistics. See also: http://www.embopress.org/page/journal/14693178/authorguide#statisticalanalysis

If n<5, please show single datapoints for diagrams.

9) Please also note our reference format:
http://www.embopress.org/page/journal/14693178/authorguide#referencesformat

10) We updated our journal's competing interests policy in January 2022 and request authors to consider both actual and perceived competing interests. Please review the policy https://www.embopress.org/competing-interests and update your competing interests if necessary. Please name this section 'Disclosure and Competing Interests Statement' and put it after the Acknowledgements section.

11) We now use CRediT to specify the contributions of each author in the journal submission system. CRediT replaces the author contribution section. Please use the free text box to provide more detailed descriptions. See also guide to authors: https://www.embopress.org/page/journal/14693178/authorguide#authorshipguidelines

12) Please add up to five keywords below the abstract and order the manuscript sections like this, using these names:
Title page - Abstract - Keywords - Introduction - Results - Discussion - Materials and Methods - Data availability section - Acknowledgements (including funding information) - Disclosure and Competing Interests Statement - References - Figure legends - Expanded View Figure legends

I look forward to seeing a revised version of your manuscript when it is ready. Please let me know if you have questions or comments regarding the revision.

Please use this link to submit your revision: https://embor.msubmit.net/cgi-bin/main.plex

Yours sincerely,

Achim Breiling
Senior Editor
EMBO Reports

Referee #1:

The paper presented by Röder and colleagues focus on Siglec proteins in chronic lymphocytic leukemia (CLL). In the mouse Siglec-G inhibits B cell receptor signaling and thus impacts on B cell development and activation. In the Eμ-TCL1 tumor model recapitulating important aspects of CLL pathogenesis, the authors demonstrate that Siglec-G ablation has a positive effect on the onset and aggressiveness of the disease, whereas overexpression of the inhibitory receptor prevents/delays CLL development. Downregulation of Siglec- 10, the human orthologue of Siglec-G, was detected in human CLL samples indicating the clinical relevance of the mouse studies. Overall, the results presented in the manuscript are well designed and conducted. The data in the mouse models confirm the hypothesis that downregulation of Siglec proteins is an oncogenic event in CLL pathogenesis. Thus, the study adds valuable insights into inhibitory receptor signaling in CLL biology and hints possible clinical implications of agonistic targeting of B cell inhibitory receptors.

The following comments/suggestions should be addressed to further strengthen the manuscript:

Major comments:
• The authors developed a novel mouse strain allowing the cell type specific overexpression of Siglec-G. In their Siglecg-R26ki/ki mb1cre mouse line Röder and colleagues focused on the B1a compartment, as the B cell population of interest for CLL development. However, as impaired antigen cell receptor signaling will impact on other B cell subtypes, the characterization of the B cell development and maturation in Siglecg-R26ki/ki mb1cre animals would be very interesting for the readers.
• In the present study TCL1 x Siglecg-R26ki/ki mb1cre mice were analyzed for CLL development. The authors may want to comment on their choice of using homozygous Siglecg-R26ki mice in the analysis. Is there a threshold for Siglec-G overexpression to suppress the CLL-like cell expansion?
Additionally, the survival curve of TCL1 x Siglecg-R26ki/ki mb1cre mice indicates that some aged animals develop signs of sickness (Figure 5G). Do these animals suffer from CLL development as well? Maybe in these CLL-clones Siglec-G overexpression was less effective?!
• The Ig repertoire analysis is elegantly showing the monoclonal expansion of the malignant cells in TCL1 x Siglecg-/- mice (Figure 3). The input population for the analysis in these animals as well as in controls is the CD19+ pool isolated from the peripheral blood. However, this approach might mask or underestimate clonal expansion in TCL1 x Siglecg-R26ki/ki mb1cre mice at week 48 characterized by a low, but still increased population of B220low CD5+ cells (Figure 6). The authors statement that "no leukemic clones developed"(Page 17) might be too optimistic.
• Regarding the intracellular flow cytometry measurements of signaling proteins downstream of the BCR: the authors might want to show these data after gating on B220low CD5+ cells (instead of the entire B220 pool). Alternatively, an additional supplementary graph showing the percentages of CLL-like cells within the B220+ cells would be sufficient to better evaluate the Siglec-G effect on BCR signaling.
• How much of the effects observed in BCR signaling between TCL1 x Siglecg-R26ki/ki and TCL1 x Siglecg-R26ki/ki mb1cre mice (Figure 8B) is related to the "non- tumor vs tumor" comparison? As the authors did not detect significant differences in TCL1 vs TCL1 x Siglecg-/- tumor samples one might consider (and discuss) it.

Minor comments:
• The roughly 10fold increase of the B220low CD5+ numbers in the TCL1 x Siglecg-R26ki/ki mice at week 36 (Figure 5C) in comparison to the corresponding week in TCL1 (Figure 2B) mice is surprising. Do the authors have any explanation?
• Please indicate the genetic background of the used mb1-cre line (as C57/BL6 and BALB/C mice are available).
• In Figure 1B the progressive expansion of the B220lowCD5+ compartment could be depicted with an additional graph in which it is possible to follow the expansion of the CLL-like population in the individual mice over time (similarly in Figure 4B).
• In Figure 2C and similarly in 5E, in the summary of B220lowCD5+ percentages in the liver, it would be more accurate to label the axis with 'leukocytes' instead of 'lymphocytes'.
• In Figure 7A in the density plot the abscissa seems to be mislabeled, otherwise the CLL-like population is CD5 negative.
• In Figure S2B the TCL1 x Siglecg-/- bars appear to be edited with variable patterns, unlike the solid purple color in the rest of the graphs throughout the manuscript.
• Please use a consistent nomenclature for the mouse strains throughout the manuscript (see page 11).
• In page 22 of the materials and methods please correct 'BZR'.
• In page 30 caption of Fig 2C: please correct 'leucocytes'.

Referee #2:

This work by Nitschke and co-workers uses genetic models to demonstrate a convincing role for Siglec-G in the development of

CLL. Both KO and over-expressing mouse models of Siglec-G are studied in the context of a mouse model of CLL. Results clearly and demonstrate that the kinetics of CLL development is inversely proportional to Siglec-G expression levels. Numerous measures are examined, including gross organ weight, survival, and cellular readouts. Moreover, it is demonstrated that Siglec-10 is down-regulated in human CLL. The manuscript is clear and well written and only requires a few considerations:

1) As this is the first study of this KI over-expressing mouse, a bit more characterization seems in order. Can the authors rule out that development impact given that MB1-Cre turns on very early in B cell development? Are lymphocyte proportions in the adult spleen roughly similar to WT mice?

2) Given the clear differences in Ca2+ flux, can they speculate why no differences were observed on downstream signaling molecules?

3) In the over-expressing mice, it was stated that they did not have enough CD5+ cells to perform cellular analyses. It would have been nice to see some readouts on the'total' B cells to complement the data have for the other models. If that data was acquired, it would be a useful addition.

Referee #3:

Major:

1. Results, page 6: "The size of these B1a cell populations in the two control mouse strains remained constant and did not increase until the age of 60 weeks (Fig.1B)." Is the increase at week 60 statistically significant? From the figure, this seems unlikely.

2. Results, page 6: "TCL1 mice showed an increase of the population of CLL-like cells (also characterized as B220low CD5+) in the blood from the age of 40 weeks, reaching a percentage of about 50% of all blood lymphocytes at week 60. In contrast, the CLL-like population in the blood of TCL1 x Siglecg-/- animals increased much earlier, already at the age of 20 weeks (on average 30 % of blood lymphocytes)." This text does not agree with the comparisons highlighted on the figure. The figure shows statistical comparisons of the two sets (WT vs Siglec6-/-; TCL1 vs TCL1x Siglec6-/-) to each other, whereas the text compares the same population to itself over time. This is the case for several other figures, e.g., Suppl Fig 1A+B; Fig 2A, B; Fig 4B).

This is very confusing. The statistical analyses relating to the text should also be provided. The comparisons in Suppl Fig 1C serve as an example.

3. Results, page 9: "This analysis revealed a significantly reduced binding of these antigens in TCL1 x Siglecg-/- mice in comparison to TCL1 littermates for most time points (Suppl Fig 3). This suggests an overall different BCR repertoire of CLL-like cells of TCL1 x Siglecg-/- mice, when compared to TCL1 mice. A similar shift in the BCR repertoire was detected previously in normal B1a cells of Siglecg-/-mice (Jellusova et al., 2010)." This finding is somewhat bothersome, as it suggests that the B cells that are being transformed in these mice are different than the standard model. Is the severity of the disease in these animals due to the development of a malignancy in B cells that do not usually transform rather than the transformation of same population a greater frequency? This is supported by the literature which I believe indicates that the major IgV gene families used by TCL1 mice and by B-1 cells are VH11 and VH12 (Baumgarth, 2011 cited; PMID: 32561570). This is compounded by the finding that these genes are identified when over expressing Siglec G (Fig 6). The authors appropriately address this issue in the Discussion but it remains a major concern.

4. Results, pages 14-15: Since the data in Figure 9B are not statistically significant, the authors should consider moving these to the Supplementary Section and bringing some of the data in Suppl Figs 6 and 7 to the main text?

5. Discussion, page 16: "The dependence of CLL on BCR signaling is not due to BCR-activating mutations in crucial signaling molecules as shown for diffuse B cell lymphoma (DLBCL) (Schmitz et al., 2018), but due to the ability of some CLL BCRs to cluster by binding to each other and in this way autonomously activating BCR signaling (Duhren-von Minden et al., 2012, Minici et al., 2017)." Although this is an accurate statement, it implies that binding of antigens other than companion BCRs is irrelevant. This is not necessarily the case as shown by in vivo studies (PMID: 25564405).

Minor:

1. The following sentence on page 4 is confusing or possibly incomplete. In particular the comment about V families. "Murine CD5+ B1a cells share features of human CD5+ CLL cells in some aspects, including expression of a restricted Ig repertoire using similar V with B6) families."

Point-by-point responses

Referee #1:

The paper presented by Röder and colleagues focus on Siglec proteins in chronic lymphocytic leukemia (CLL). In the mouse Siglec-G inhibits B cell receptor signaling and thus impacts on B cell development and activation. In the Eμ-TCL1 tumor model recapitulating important aspects of CLL pathogenesis, the authors demonstrate that Siglec-G ablation has a positive effect on the onset and aggressiveness of the disease, whereas overexpression of the inhibitory receptor prevents/delays CLL development. Downregulation of Siglec- 10, the human orthologue of Siglec-G, was detected in human CLL samples indicating the clinical relevance of the mouse studies. Overall, the results presented in the manuscript are well designed and conducted. The data in the mouse models confirm the hypothesis that downregulation of Siglec proteins is an oncogenic event in CLL pathogenesis. Thus, the study adds valuable insights into inhibitory receptor signaling in CLL biology and hints possible clinical implications of agonistic targeting of B cell inhibitory receptors.

The following comments/suggestions should be addressed to further strengthen the manuscript:

Major comments:

• The authors developed a novel mouse strain allowing the cell type specific overexpression of Siglec-G. In their Siglecg-R26ki/ki mb1cre mouse line Röder and colleagues focused on the B1a compartment, as the B cell population of interest for CLL development. However, as impaired antigen cell receptor signaling will impact on other B cell subtypes, the characterization of the B cell development and maturation in Siglecg-R26ki/ki mb1cre animals would be very interesting for the readers.

Response
We thank the reviewer for the positive comments to our manuscript. We agree with the suggestion that a characterisation of the new SiglecG overexpressing mouse line in more detail is helpful for the reader. We have therefore performed more experiments to analyse this in detail. We find no impairment of B cell development or B cell subpopulations in the spleen in Siglecg-R26ki/ki mb1cre mice. These data are now shown in the new Fig EV 6. The only detected difference of this mouse line is a lower number of B1a cells (shown in Fig. EV 5).

• In the present study TCL1 x Siglecg-R26ki/ki mb1cre mice were analyzed for CLL development. The authors may want to comment on their choice of using homozygous Siglecg-R26ki mice in the analysis. Is there a threshold for Siglec-G overexpression to suppress the CLL-like cell expansion?

Response
The homozygous Siglecg-R26ki mice show an overexpression level of Siglec-G which is about 5 fold higher on B cells than in WT control mice. We chose this model, because we felt it still is a moderate higher expression pattern. Heterozygous mice were not tested in the TCL1 model, although it would be an interesting question to address. Since these long-term experiments for 1 year already involved 4 groups of mice, we did not want to include a further group. Therefore, we have not addressed this question.

Additionally, the survival curve of TCL1 x Siglecg-R26ki/ki mb1cre mice indicates that some aged animals develop signs of sickness (Figure 5G). Do these animals suffer from CLL development as well? Maybe in these CLL-clones Siglec-G overexpression was less effective?!

Response
We can only speculate why some of the TCL1 x Siglecg-R26ki/ki mb1cre mice were also affected by a slightly lower survival. We have added one new figure (Fig. EV 1B), which shows the CLL progression in individual mice. In this figure you can see that the CLL development in this group is heterogenous. While most mice stay down with hardly any CLL-like cells in the blood until week 48, one or two mice develop some CLL cells, but to a lower cellular percentage than most controls.

• The Ig repertoire analysis is elegantly showing the monoclonal expansion of the malignant cells in

TCL1 x Siglecg-/- mice (Figure 3). The input population for the analysis in these animals as well as in controls is the CD19+ pool isolated from the peripheral blood. However, this approach might mask or underestimate clonal expansion in TCL1 x Siglecg-R26ki/ki mb1cre mice at week 48 characterized by a low, but still increased population of B220low CD5+ cells (Figure 6). The authors statement that "no leukemic clones developed"(Page 17) might be too optimistic.

Response
Yes, we agree and added the new figure (Fig. EV 1B), which shows the CLL progression in individual mice detects rising CLL-like populations in at least a few TCL1 x Siglecg-R26ki/ki mb1cre mice. We have modified the sentence on page 17 to: “no leukemic clones were detected by our analysis”.

• Regarding the intracellular flow cytometry measurements of signaling proteins downstream of the BCR: the authors might want to show these data after gating on B220low CD5+ cells (instead of the entire B220 pool). Alternatively, an additional supplementary graph showing the percentages of CLL-like cells within the B220+ cells would be sufficient to better evaluate the Siglec-G effect on BCR signaling.
• How much of the effects observed in BCR signaling between TCL1 x Siglecg-R26ki/ki and TCL1 x Siglecg-R26ki/ki mb1cre mice (Figure 8B) is related to the "non- tumor vs tumor" comparison? As the authors did not detect significant differences in TCL1 vs TCL1 x Siglecg-/- tumor samples one might consider (and discuss) it.

Response
The suggestion of the reviewer to gate on B220low CD5+ cells was difficult to do, as this population was so small in the TCL1 x Siglecg-R26ki/ki mb1cre mice. However, we have added a supplementary graph showing the CLL-like cells of the TCL1 x Siglecg-R26ki/ki and TCL1 x Siglecg-R26ki/ki mb1cre mice of Figure 8B (new Fig EV 8). We have described this in the results section: “Although the percentage of CLL-like cells analyzed in Fig.8B was on average not different between TCL1 x *Siglecg*-R26$^{ki/ki}$ and TCL1 x *Siglecg*-R26$^{ki/ki}$ x mb1^{cre} mice, the latter had more widespread CLL-like numbers (Fig. EV 8). Therefore, their normal phopho-protein levels maybe also due to the higher percentage of normal B cells” (page 14).

Minor comments:
• The roughly 10fold increase of the B220low CD5+ numbers in the TCL1 x Siglecg-R26ki/ki mice at week 36 (Figure 5C) in comparison to the corresponding week in TCL1 (Figure 2B) mice is surprising. Do the authors have any explanation?
• Please indicate the genetic background of the used mb1-cre line (as C57/BL6 and BALB/C mice are available).

Response
The stronger (and earlier) increase of CLL-like cells of the blood, spleen and other organs in controls of the second cohort (TCL1 x Siglecg-R26ki/ki mice), compared to the controls of the first cohort (TCL1 mice) was also noted by us. We cannot explain this, as all mice were on a pure BALB/c background. This information is now also added for the mb1-cre line in materials and methods. But of course, the TCL1 x Siglecg-R26ki/ki mice are the proper control for the same line with the additional mb1-cre allele, whereas the TCL1 line (on BALB/c) is the proper control for *Siglecg*-/- (on BALB/c). It is noticeable, but not easy to explain.

• In Figure 1B the progressive expansion of the B220lowCD5+ compartment could be depicted with an additional graph in which it is possible to follow the expansion of the CLL-like population in the individual mice over time (similarly in Figure 4B).

Response
Yes, as mentioned previously, these data are now shown in the new Fig. EV 1.

• In Figure 2C and similarly in 5E, in the summary of B220lowCD5+ percentages in the liver, it would be more accurate to label the axis with 'leukocytes' instead of 'lymphocytes'.
• In Figure 7A in the density plot the abscissa seems to be mislabeled, otherwise the CLL-like population is CD5 negative.
• In Figure S2B the TCL1 x Siglecg-/- bars appear to be edited with variable patterns, unlike the solid

purple color in the rest of the graphs throughout the manuscript.
• Please use a consistent nomenclature for the mouse strains throughout the manuscript (see page 11).
• In page 22 of the materials and methods please correct 'BZR'.
• In page 30 caption of Fig 2C: please correct 'leucocytes'.

Response
All these points were corrected in the new manuscript. For Fig. 7A, we chose a new example. Due to the UV laser needed for Ca2+ measurements, another fluorochrome on the anti-CD5 antibody had to be chosen than in all other figures, which separates CD5-low cells not so well from CD5-negative cells.

Referee #2:

This work by Nitschke and co-workers uses genetic models to demonstrate a convincing role for Siglec-G in the development of CLL. Both KO and over-expressing mouse models of Siglec-G are studied in the context of a mouse model of CLL. Results clearly and demonstrate that the kinetics of CLL development is inversely proportional to Siglec-G expression levels. Numerous measures are examined, including gross organ weight, survival, and cellular readouts. Moreover, it is demonstrated that Siglec-10 is down-regulated in human CLL. The manuscript is clear and well written and only requires a few considerations:

1) As this is the first study of this KI over-expressing mouse, a bit more characterization seems in order. Can the authors rule out that development impact given that MB1-Cre turns on very early in B cell development? Are lymphocyte proportions in the adult spleen roughly similar to WT mice?

Response
We thank the reviewer for the positive comments. As in response to reviewer no.1, we agree with the suggestion that a characterisation of the new SiglecG overexpressing mouse line in more detail is helpful for the reader. We have therefore performed more experiments to analyse this in detail. We find no impairment of B cell development or B cell subpopulations in the spleen in Siglecg-R26ki/ki mb1cre mice. These data are now shown in the new Fig EV 6. The only detected difference of this mouse line is a lower number of B1a cells (shown in Fig. EV 5).

2) Given the clear differences in Ca2+ flux, can they speculate why no differences were observed on downstream signaling molecules?

Response
Yes, it is surprising that we cannot detect differences particularly in the phospho-proteins of the proximal signalling pathways between TCL1 and TCL1 x *Siglecg-/-* CLL-like cells. This has also been the case for the original description of the *Siglecg-/-* mice, where we detected the largely increased Ca2+ signalling in B1a cells, but could not find obvious increases in proximal signalling pathways in B1a cells by phospho-FACS (Hoffmann et al. Nat.Immunol.2007). So the TCL1 mice reflect also the normal *Siglecg-/-* mice. It maybe that we did not look at the right intracellular signalling proteins. For this, phopho-protein analysis is limited.

3) In the over-expressing mice, it was stated that they did not have enough CD5+ cells to perform cellular analyses. It would have been nice to see some readouts on the 'total' B cells to complement the data have for the other models. If that data was acquired, it would be a useful addition.

Response
Although we tried to do these additional experiments, we did not obtain conclusive results. Maybe because the total B cells are varying too much in their composition from mouse to mouse. We have added a sentence to the results section describing this: “Attempts to measure BCR-induced Ca^{2+} responses in Siglec-G overexpressing mice were not successful due to the very low number of $B220^{low}CD5^{+}$ cells in *Siglecg*-$R26^{ki/ki}$ $mb1^{cre}$ and TCL1 x *Siglecg*-$R26^{ki/ki}$ $mb1^{cre}$ mice and due to inconsistent results when gating on total B cells”.

Referee #3:

Major:
1. Results, page 6: "The size of these B1a cell populations in the two control mouse strains remained constant and did not increase until the age of 60 weeks (Fig.1B)." Is the increase at week 60 statistically significant? From the figure, this seems unlikely.

Response
We thank the reviewer for the comments. The increase at week 60 between the two control mouse strains is not significant. All significant differences within the time points are shown.

2. Results, page 6: "TCL1 mice showed an increase of the population of CLL-like cells (also characterized as B220low CD5+) in the blood from the age of 40 weeks, reaching a percentage of about 50% of all blood lymphocytes at week 60. In contrast, the CLL-like population in the blood of TCL1 x Siglecg-/- animals increased much earlier, already at the age of 20 weeks (on average 30 % of blood lymphocytes)." This text does not agree with the comparisons highlighted on the figure. The figure shows statistical comparisons of the two sets (WT vs Siglec6-/-; TCL1 vs TCL1x Siglec6-/-) to each other, whereas the text compares the same population to itself over time. This is the case for several other figures, e.g., Suppl Fig 1A+B; Fig 2A, B; Fig 4B).

This is very confusing. The statistical analyses relating to the text should also be provided. The comparisons in Suppl Fig 1C serve as an example.

Response
We only show significant differences within each time point between the different mouse strains. We do not show differences over time, as this would be too confusing to show as well. We agree that the discussion of the findings in the results section was a bit focused on the changes during time. We have now changed the description of the results in all the respective parts. All the changes are marked in yellow. Parts describing the changes in time were deleted or shortened and more emphasis is now on the differences within the groups of mice.

3. Results, page 9: "This analysis revealed a significantly reduced binding of these antigens in TCL1 x Siglecg-/- mice in comparison to TCL1 littermates for most time points (Suppl Fig 3). This suggests an overall different BCR repertoire of CLL-like cells of TCL1 x Siglecg-/- mice, when compared to TCL1 mice. A similar shift in the BCR repertoire was detected previously in normal B1a cells of Siglecg-/- mice (Jellusova et al., 2010)." This finding is somewhat bothersome, as it suggests that the B cells that are being transformed in these mice are different than the standard model. Is the severity of the disease in these animals due to the development of a malignancy in B cells that do not usually transform rather than the transformation of same population a greater frequency? This is supported by the literature which I believe indicates that the major IgV gene families used by TCL1 mice and by B-1 cells are VH11 and VH12 (Baumgarth, 2011 cited; PMID: 32561570). This is compounded by the finding that these genes are identified when over expressing Siglec G (Fig 6). The authors appropriately address this issue in the Discussion but it remains a major concern.

Response
The group of Chiorazzi has analysed the CLL clones in TCL1 mice in detail (Yan et al 2006, cited in our paper). VH11 and VH12 were used more often in these clones than their normal representation in the Ig repertoire, but both in about 10% of the CLL clones. This means that 80% of the CLL clones contained other VH families. Therefore, we would argue that the shift in the Ig repertoire of CLL cells in TCL1 x Siglecg-/- mice is interesting, but does not necessarily mean that it is a different way of transformation. But it sheds light on the point that not only the VH11 and VH12 clones are the crucial VH families for CLL transformation. To clarify this point we have now added a sentence in the discussion: “Similarly, over-usage of the V_H11, V_H12, and V_H4 gene segments has been demonstrated in TCL1 animals. Each of these three V_H families was used in about 10% of leukemic clones of TCL1 transgenic mice, while the rest of leukemic clones used other V_H families (Yan et al., 2006)“ (page 18).

4. Results, pages 14-15: Since the data in Figure 9B are not statistically significant, the authors should consider moving these to the Supplementary Section and bringing some of the data in Suppl Figs 6 and 7 to the main text?

Response
Thank you for this suggestion, yes, we removed the data from Fig. 9B to the supplementary data and brought one figure from the supplementary data to the main Fig. 9B (differential mRNA expression).

5. Discussion, page 16: "The dependence of CLL on BCR signaling is not due to BCR-activating mutations in crucial signaling molecules as shown for diffuse B cell lymphoma (DLBCL) (Schmitz et al., 2018), but due to the ability of some CLL BCRs to cluster by binding to each other and in this way autonomously activating BCR signaling (Duhren-von Minden et al., 2012, Minici et al., 2017)." Although this is an accurate statement, it implies that binding of antigens other than companion BCRs is irrelevant. This is not necessarily the case as shown by in vivo studies (PMID: 25564405).

Response
Thank you for this comment. We have added the ref. which you mentioned and changed the statement accordingly: "The induction of BCR signals by low affinity autoantigens also plays a role in CLL development (Iacovelli et al. 2015)". (page 14)

Minor:
1. The following sentence on page 4 is confusing or possibly incomplete. In particular the comment about V families. "Murine CD5+ B1a cells share features of human CD5+ CLL cells in some aspects, including expression of a restricted Ig repertoire using similar V with B6) families."

Response
This was corrected.

1st Revision - Editorial Decision 27th Apr 2023

Dear Prof. Nitschke,

Thank you for the submission of your revised manuscript to our editorial offices. I have now received the reports from the three referees that I asked to re-evaluate your study, you will find below. As you will see, the referees now fully support the publication of your study in EMBO reports.

Before proceeding with formal acceptance, I have these editorial requests I ask you to address in a final revised manuscript:

- Please provide the abstract written in present tense.

- The figures of the manuscript need to be re-organized, and we need single figure files also for the EV figures. The Expanded View format, which will be displayed in the main HTML of the paper in a collapsible format, has replaced the Supplementary information. You can submit up to 5 images as Expanded View. Presently, there are 10!

Looking through the figures, I think several figures could be combined. I would suggest doing that to have 6-7 (max. 8) main figures and 5 EV figures. Please follow the nomenclature Figure EV1, Figure EV2 etc. The figure legend for these should be included in the main manuscript document file in a section called Expanded View Figure Legends after the main Figure Legends section.

Remaining or additional Supplementary material would need to be supplied as a single pdf file labeled Appendix. The Appendix should have page numbers and needs to include a table of content on the first page (with page numbers) and legends for all content. Please follow the nomenclature Appendix Figure Sx, Appendix Table Sx etc. throughout the text, and also label the figures and tables according to this nomenclature.

For more details, please refer to our guide to authors:
http://www.embopress.org/page/journal/14693178/authorguide#manuscriptpreparation

Please consult our guide for figure preparation:
http://wol-prod-cdn.literatumonline.com/pb-assets/embo-site/EMBOPress_Figure_Guidelines_061115-1561436025777.pdf

See also the guidelines for figure legend preparation:
https://www.embopress.org/page/journal/14693178/authorguide#figureformat

Finally, please update all the figure callouts!

- Please also upload Table EV1 as individual file using the file type 'Expanded View' (or add this to an Appendix file).

- We now use CRediT to specify the contributions of each author in the journal submission system. CRediT replaces the author contribution section. Please use the free text box to provide more detailed descriptions and remove the author contributions section from the manuscript text file. See also guide to authors:
https://www.embopress.org/page/journal/14693178/authorguide#authorshipguidelines

- We updated our journal's competing interests policy in January 2022 and request authors to consider both actual and perceived competing interests. Please review the policy https://www.embopress.org/competing-interests and update your competing interests if necessary. Please name this section 'Disclosure and Competing Interests Statement' and put it after the Acknowledgements section.

- We could not find the source data for Fig. 7. Please provide the information on the deposited dataset for Fig. 7A in the data availability section and upload the SD for Fig. 7B.

- Please remove the referee token from the data availability section and make sure that the data is public latest when the manuscript is published online.

- Please order the manuscript sections like this (using these names as headings):
Title page - Abstract - Keywords - Introduction - Results - Discussion - Materials and Methods - Data availability section - Acknowledgements - Disclosure and Competing Interests Statement - References - Figure legends - Expanded View Figure Legends

- Regarding data quantification and statistics, please make sure that the number "n" for how many independent experiments were performed, their nature (biological versus technical replicates), the bars and error bars (e.g. SEM, SD) and the test used to calculate p-values is indicated in the respective figure legends (also for potential EV figures and all those in the final Appendix). Please also check that all the p-values are explained in the legend, and that these fit to those shown in the figure. Please

provide statistical testing where applicable. Please avoid the phrase 'independent experiment', but clearly state if these were biological or technical replicates. Please also indicate (e.g. with n.s.) if testing was performed, but the differences are not significant. In case n=2 please show the data as separate datapoints or bars without error bars and statistics. See also: http://www.embopress.org/page/journal/14693178/authorguide#statisticalanalysis

If n<5, please show single datapoints for diagrams. Presently, there are still diagrams without or with only partial statistics (missing e.g. n.s.). Please fix this.

- Please make sure that all figure panels are called out separately and sequentially (main, EV and Appendix figures). Presently, there are no separate callouts for Figs. EV3A&B and EV4A&B. Please check.

- Please provide the author checklist with answers, i.e. select responses from the drop-down lists.

- Finally, please find attached a word file of the manuscript text (provided by our publisher) with changes we ask you to include in your final manuscript text and comments. Please use the attached file as basis for further revisions and provide your final manuscript file with track changes, in order that we can see any modifications done.

In addition, I would need from you:
- a short, two-sentence summary of the manuscript (not more than 35 words).
- two to four short bullet points highlighting the key findings of your study (two lines each).
- a schematic summary figure as separate file that provides a sketch of the major findings (not a data image) in jpeg or tiff format (with the exact width of 550 pixels and a height of not more than 400 pixels) that can be used as a visual synopsis on our website.

I look forward to seeing the final revised version of your manuscript when it is ready. Please let me know if you have questions regarding the revision.

Please use this link to submit your revision: https://embor.msubmit.net/cgi-bin/main.plex

Best,

Achim Breiling
Senior Editor
EMBO Reports

Referee #1:

Thank you for the revised manuscript. I have no additional comments.

Referee #2:

The authors have satisfactorily addressed my previous comments

----
Referee #3:

The manuscript is suitable for publication in EMBO reports without further revision.

The authors have addressed all minor editorial requests.

Prof. Lars Nitschke
University of Erlangen
Chair of Genetics, Department of Biology
Staudtstr. 5
Erlangen 91058
Germany

Dear Prof. Nitschke,

I am very pleased to accept your manuscript for publication in the next available issue of EMBO reports. Thank you for your contribution to our journal.

At the end of this email I include important information about how to proceed. Please ensure that you take the time to read the information and complete and return the necessary forms to allow us to publish your manuscript as quickly as possible.

As part of the EMBO publication's Transparent Editorial Process, EMBO reports publishes online a Review Process File to accompany accepted manuscripts. As you are aware, this File will be published in conjunction with your paper and will include the referee reports, your point-by-point response and all pertinent correspondence relating to the manuscript.

If you do NOT want this File to be published, please inform the editorial office within 2 days, if you have not done so already, otherwise the File will be published by default [contact: emboreports@embo.org]. If you do opt out, the Review Process File link will point to the following statement: "No Review Process File is available with this article, as the authors have chosen not to make the review process public in this case."

Thank you again for your contribution to EMBO reports and congratulations on a successful publication. Please consider us again in the future for your most exciting work.

Yours sincerely,

Achim Breiling
Senior Editor
EMBO Reports

****

THINGS TO DO NOW:

Please note that you will be contacted by Wiley Author Services to complete licensing and payment information. The required 'Page Charges Authorization Form' is available here: https://www.embopress.org/pb-assets/embo-site/er_apc.pdf - please download and complete the form and return to embopressproduction@wiley.com

EMBO Press participates in many Publish and Read agreements that allow authors to publish Open Access with reduced/no publication charges. Check your eligibility: https://authorservices.wiley.com/author-resources/Journal-Authors/open-access/affiliation-policies-payments/index.html

You will receive proofs by e-mail approximately 2-3 weeks after all relevant files have been sent to our Production Office; you should return your corrections within 2 days of receiving the proofs.

Please inform us if there is likely to be any difficulty in reaching you at the above address at that time. Failure to meet our deadlines may result in a delay of publication, or publication without your corrections.

All further communications concerning your paper should quote reference number EMBOR-2022-56420V3 and be addressed to emboreports@wiley.com.

Should you be planning a Press Release on your article, please get in contact with emboreports@wiley.com as early as possible, in order to coordinate publication and release dates.

EMBO Press Author Checklist

Corresponding Author Name: Prof. Lars Nitschke	
Journal Submitted to: EMBO reports	
Manuscript Number: EMBOR-2022-56420V2	

USEFUL LINKS FOR COMPLETING THIS FORM
The EMBO Journal - Author Guidelines
EMBO Reports - Author Guidelines
Molecular Systems Biology - Author Guidelines
EMBO Molecular Medicine - Author Guidelines

Reporting Checklist for Life Science Articles (updated January 2022)
This checklist is adapted from Materials Design Analysis Reporting (MDAR) Checklist for Authors. MDAR establishes a minimum set of requirements in transparent reporting in the life sciences (see Statement of Task: 10.31222/osf.io/9sm4x). Please follow the journal's guidelines in preparing your manuscript.
Please note that a copy of this checklist will be published alongside your article.

Abridged guidelines for figures
1. Data
The data shown in figures should satisfy the following conditions:
- the data were obtained and processed according to the field's best practice and are presented to reflect the results of the experiments in an accurate and unbiased manner.
- ideally, figure panels should include only measurements that are directly comparable to each other and obtained with the same assay.
- plots include clearly labeled error bars for independent experiments and sample sizes. Unless justified, error bars should not be shown for technical replicates.
- if n<5, the individual data points from each experiment should be plotted. Any statistical test employed should be justified.
- Source Data should be included to report the data underlying figures according to the guidelines set out in the authorship guidelines on Data Presentation.

2. Captions
Each figure caption should contain the following information, for each panel where they are relevant:
- a specification of the experimental system investigated (eg cell line, species name).
- the assay(s) and method(s) used to carry out the reported observations and measurements.
- an explicit mention of the biological and chemical entity(ies) that are being measured.
- an explicit mention of the biological and chemical entity(ies) that are altered/varied/perturbed in a controlled manner.
- the exact sample size (n) for each experimental group/condition, given as a number, not a range;
- a description of the sample collection allowing the reader to understand whether the samples represent technical or biological replicates (including how many animals, litters, cultures, etc.).
- a statement of how many times the experiment shown was independently replicated in the laboratory.
- definitions of statistical methods and measures:
  - common tests, such as t-test (please specify whether paired vs. unpaired), simple $\chi 2$ tests, Wilcoxon and Mann-Whitney tests, can be unambiguously identified by name only, but more complex techniques should be described in the methods section;
  - are tests one-sided or two-sided?
  - are there adjustments for multiple comparisons?
  - exact statistical test results, e.g., P values = x but not P values < x;
  - definition of 'center values' as median or average;
  - definition of error bars as s.d. or s.e.m.

Please complete ALL of the questions below.
Select "Not Applicable" only when the requested information is not relevant for your study.

Materials

Newly Created Materials	Information included in the manuscript?	In which section is the information available? (Reagents and Tools Table, Materials and Methods, Figures, Data Availability Section)
New materials and reagents need to be available; do any restrictions apply?	Yes	there are no restrictions, section Materials and Methods

Antibodies	Information included in the manuscript?	In which section is the information available? (Reagents and Tools Table, Materials and Methods, Figures, Data Availability Section)
For **antibodies** provide the following information:<br>- Commercial antibodies: RRID (if possible) or supplier name, catalogue number and or/clone number<br>- Non-commercial: RRID or citation	Yes	Materials and Methods

DNA and RNA sequences	Information included in the manuscript?	In which section is the information available? (Reagents and Tools Table, Materials and Methods, Figures, Data Availability Section)
**Short novel DNA or RNA including primers, probes**: provide the sequences.	Yes	Materials and Methods

Cell materials	Information included in the manuscript?	In which section is the information available? (Reagents and Tools Table, Materials and Methods, Figures, Data Availability Section)
**Cell lines:** Provide species information, strain. Provide accession number in repository **OR** supplier name, catalog number, clone number, and/**OR** RRID.	Not Applicable	not applicable
**Primary cultures:** Provide species, strain, sex of origin, genetic modification status.	Not Applicable	not applicable
Report if the cell lines were recently **authenticated** (e.g., by STR profiling) and tested for mycoplasma contamination.	Not Applicable	not applicable

Experimental animals	Information included in the manuscript?	In which section is the information available? (Reagents and Tools Table, Materials and Methods, Figures, Data Availability Section)
**Laboratory animals or Model organisms:** Provide species, strain, sex, age, genetic modification status. Provide accession number in repository **OR** supplier name, catalog number, clone number, **OR** RRID.	Yes	Materials and Methods
**Animal observed in or captured from the field:** Provide species, sex, and age where possible.	Not Applicable	not applicable
Please detail **housing and husbandry conditions**.	Yes	Materials and Methods

Plants and microbes	Information included in the manuscript?	In which section is the information available? (Reagents and Tools Table, Materials and Methods, Figures, Data Availability Section)
**Plants:** provide species and strain, ecotype and cultivar where relevant, unique accession number if available, and source (including location for collected wild specimens).	Not Applicable	not applicable
**Microbes:** provide species and strain, unique accession number if available, and source.	Not Applicable	not applicable

Human research participants	Information included in the manuscript?	In which section is the information available? (Reagents and Tools Table, Materials and Methods, Figures, Data Availability Section)
If collected and within the bounds of privacy constraints report on age, sex and gender or ethnicity for all study participants.	Yes	Materials and Methods

Core facilities	Information included in the manuscript?	In which section is the information available? (Reagents and Tools Table, Materials and Methods, Figures, Data Availability Section)
If your work benefited from core facilities, was their service mentioned in the acknowledgments section?	Yes	Materials and Methods

Design

Study protocol	Information included in the manuscript?	In which section is the information available? (Reagents and Tools Table, Materials and Methods, Figures, Data Availability Section)
If study protocol has been **pre-registered, provide DOI in the manuscript**. For clinical trials, provide the trial registration number **OR** cite DOI.	Not Applicable	not applicable
Report the **clinical trial registration number** (at ClinicalTrials.gov or equivalent), where applicable.	Not Applicable	not applicable

Laboratory protocol	Information included in the manuscript?	In which section is the information available? (Reagents and Tools Table, Materials and Methods, Figures, Data Availability Section)
Provide DOI OR other citation details if **external detailed step-by-step protocols** are available.	Not Applicable	not applicable

Experimental study design and statistics	Information included in the manuscript?	In which section is the information available? (Reagents and Tools Table, Materials and Methods, Figures, Data Availability Section)
Include a statement about **sample size** estimate even if no statistical methods were used.	Yes	Materials and Methods
Were any steps taken to minimize the effects of subjective bias when allocating animals/samples to treatment (e.g. **randomization procedure**)? If yes, have they been described?	Not Applicable	not applicable
Include a statement about **blinding** even if no blinding was done.	Yes	no blinding was done
Describe **inclusion/exclusion criteria** if samples or animals were excluded from the analysis. Were the criteria pre-established?<br><br>If sample or data points were omitted from analysis, report if this was due to attrition or intentional exclusion and provide justification.	Not Applicable	not applicable
For every figure, are **statistical tests** justified as appropriate? Do the data meet the assumptions of the tests (e.g., normal distribution)? Describe any methods used to assess it. Is there an estimate of variation within each group of data? Is the variance similar between the groups that are being statistically compared?	Yes	all statistical tests were done according to the set of data with the relevant test for normal distribution or non-normal distribution. The details are given in the legends of the figures.

Sample definition and in-laboratory replication	Information included in the manuscript?	In which section is the information available? (Reagents and Tools Table, Materials and Methods, Figures, Data Availability Section)
In the figure legends: state number of times the experiment was **replicated** in laboratory.	Yes	in the fig legends
In the figure legends: define whether data describe **technical or biological replicates**.	Yes	yes, given in the fig legends

Ethics

Ethics	Information included in the manuscript?	In which section is the information available? (Reagents and Tools Table, Materials and Methods, Figures, Data Availability Section)
Studies involving **human participants**: State details of **authority granting ethics approval** (IRB or equivalent committee(s), provide reference number for approval.	Yes	Materials and Methods
Studies involving **human participants**: Include a statement confirming that **informed consent** was obtained from all subjects and that the experiments conformed to the principles set out in the WMA Declaration of Helsinki and the Department of Health and Human Services Belmont Report.	Yes	Materials and Methods
Studies involving **human participants:** For publication of **patient photos**, include a statement confirming that consent to publish was obtained.	Not Applicable	not applicable
Studies involving experimental **animals**: State details of **authority granting ethics approval** (IRB or equivalent committee(s), provide reference number for approval. Include a statement of compliance with ethical regulations.	Yes	Materials and Methods
Studies involving **specimen and field samples:** State if relevant **permits** obtained, provide details of authority approving study; if none were required, explain why.	Not Applicable	not applicable

Dual Use Research of Concern (DURC)	Information included in the manuscript?	In which section is the information available? (Reagents and Tools Table, Materials and Methods, Figures, Data Availability Section)
Could your study fall under dual use research restrictions? Please check biosecurity documents and list of **select agents and toxins** (CDC): https://www.selectagents.gov/sat/list.htm	Not Applicable	not applicable
If you used a select agent, is the security level of the lab appropriate and reported in the manuscript?	Not Applicable	not applicable
If a study is subject to dual use research of concern regulations, is the name of the **authority granting approval and reference number** for the regulatory approval provided in the manuscript?	Not Applicable	not applicable

Reporting

The MDAR framework recommends adoption of discipline-specific guidelines, established and endorsed through community initiatives. Journals have their own policy about requiring specific guidelines and recommendations to complement MDAR.

Adherence to community standards	Information included in the manuscript?	In which section is the information available? (Reagents and Tools Table, Materials and Methods, Figures, Data Availability Section)
State if relevant guidelines or checklists (e.g., **ICMJE, MIBBI, ARRIVE, PRISMA**) have been followed or provided.	Not Applicable	not applicable
For **tumor marker prognostic studies,** we recommend that you follow the **REMARK** reporting guidelines (see link list at top right). See author guidelines, under 'Reporting Guidelines'. Please confirm you have followed these guidelines.	Not Applicable	not applicable
For **phase II and III randomized controlled trials**, please refer to the **CONSORT** flow diagram (see link list at top right) and submit the CONSORT checklist (see link list at top right) with your submission. See author guidelines, under 'Reporting Guidelines'. Please confirm you have submitted this list.	Not Applicable	not applicable

Data Availability

Data availability	Information included in the manuscript?	In which section is the information available? (Reagents and Tools Table, Materials and Methods, Figures, Data Availability Section)
Have **primary datasets** been deposited according to the journal's guidelines (see 'Data Deposition' section) and the respective accession numbers provided in the Data Availability Section?	Yes	Data Availability Section
Were **human clinical and genomic datasets** deposited in a public access-controlled repository in accordance to ethical obligations to the patients and to the applicable consent agreement?	Not Applicable	not applicable
Are **computational models** that are central and integral to a study available without restrictions in a machine-readable form? Were the relevant accession numbers or links provided?	Not Applicable	not applicable
If publicly available data were reused, provide the respective **data citations in the reference list**.	Not Applicable	not applicable
